# Pulmonary and extra-pulmonary infections caused by classical and hypervirulent *Klebsiella pneumoniae*: a prospective cross-sectional study

**DOI:** 10.3389/fmicb.2025.1707017

**Published:** 2026-01-02

**Authors:** Fatma Alshehri, Rasha A. Mosbah, Sozan M. Abdelkhalig, Noaf Abdullah N. Alblwi, Shereen Fawzy, Hanan Alshareef, Rehab Ahmed, Sawsan A. Zaitone, Abir S. Mohamed, Abdallah Tageldein Mansour, Majid Alhomrani, Abdulhakeem S. Alamri, Mahmoud M. Bendary

**Affiliations:** 1 Department of Biology, College of Sciences, Princess Nourah bint Abdulrahman University, Riyadh, Saudi Arabia; 2 Infection Control Unit, Zagazig University Hospital, Zagazig, Egypt; 3 Military Medical Academy, Cairo, Egypt; 4 Department of Basic Medical Sciences, College of Medicine, AlMaarefa University, Riyadh, Saudi Arabia; 5 Research Center, Deanship of Scientific Research and Post-Graduate Studies, AlMaarefa University, Riyadh, Saudi Arabia; 6 Al Hadithah General Hospital, Al-Qurayyat, Saudi Arabia; 7 Department of Medical Microbiology, Faculty of Medicine, University of Tabuk, Tabuk, Saudi Arabia; 8 Department of Pharmacy Practice, Faculty of Pharmacy, University of Tabuk, Tabuk, Saudi Arabia; 9 Division of Microbiology, Immunology and Biotechnology, Department of Natural Products and Alternative Medicine, Faculty of Pharmacy, University of Tabuk, Tabuk, Saudi Arabia; 10 Department of Pharmacology and Toxicology, Faculty of Pharmacy, University of Tabuk, Tabuk, Saudi Arabia; 11 Department of Public Health, College of Nursing and Health Sciences, Jazan University, Jazan, Saudi Arabia; 12 Department of Fish and Animal Production, College of Agriculture and Food Sciences, King Faisal University, Al-Ahsa, Saudi Arabia; 13 Department of Clinical Laboratories Sciences, The Faculty of Applied Medical Science, Taif University, Taif, Saudi Arabia; 14 Centre of Biomedical Science Research (CBSR), Deanship of Scientific Research, Taif University, Taif, Saudi Arabia; 15 Department of Microbiology and Immunology, Faculty of Pharmacy, Port Said University, Port Said, Egypt

**Keywords:** *Klebsiella pneumoniae*, classical (cKp), hypervirulent (hvKp), multidrug resistance (MDR), carbapenem-resistant (CRKP), pulmonary infections, extrapulmonary infections

## Abstract

**Aim:**

*Klebsiella pneumoniae* (*K. pneumoniae*) is an opportunistic pathogen causing infections ranging from pulmonary disease to severe systemic illness. This study aimed to examine the relationships between infection site, pathotype, virulence, resistance, and clinical presentations to manage classical (cKp) and hypervirulent (hvKp) strains.

**Methods and results:**

600 clinical specimens were screened for *K. pneumoniae*. Virulence and resistance fitness were analyzed phenotypically and genotypically. Clinical correlations were assessed to link pathotype, resistance, virulence, infection site, age, and presentation. *K. pneumoniae* was detected in 17.2% specimens (103/600), with pulmonary samples showing 43% positivity versus 12% in extra-pulmonary ones. hvKp accounted for 55.3% and cKp for 44.7% of isolates; hvKp predominated in extra-pulmonary infections (83.3%) and cKp in pulmonary cases (83.7%). K1 (33.1%) and K2 (21.4%) were confined to hvKp, while pulmonary isolates were mainly K3, K5, and K20. Pulmonary infections mainly affected adults (81.4%) and showed respiratory signs; extra-pulmonary cases occurred mostly in pediatric (46.7%) and elderly (41.7%) patients with systemic symptoms such as fever and hypotension. Clustering distinguished pulmonary/cKp (MDR-rich) from extra-pulmonary/hvKp (virulence-rich) isolates, with some overlap indicating hybrid strains. Correlations linked pulmonary infections with cKp and MDR (r = 0.67), and extra-pulmonary infections with hvKp and systemic disease (r = 0.67).

**Conclusion:**

The high MDR burden of cKp underscores the need for stronger antimicrobial stewardship and new therapies, while the invasive nature of hvKp highlights the importance of early recognition and rapid intervention to prevent systemic complications. Early distinction of cKp from hvKp can significantly influence treatment decisions and reduce morbidity and mortality associated with *K. pneumoniae* infections.

## Introduction

*Klebsiella pneumoniae* (*K. pneumoniae*) is a Gram-negative, capsule-forming, non-motile bacterium in the Enterobacteriaceae family. It is a major cause of nosocomial and community-acquired infections. Once viewed mainly as a hospital pathogen, it is now known to be globally distributed across humans, animals, and environments such as soil, water, plants, and food (De Koster et al., 2022; Elmanakhly et al., 2022). Its wide presence supports gene exchange for resistance and virulence, aiding persistence worldwide (Wareth and Neubauer, 2021). Reports confirm its spread in over 57 countries, affecting humans, livestock, and companion animals (Liu et al., 2024). *K. pneumoniae* causes 20–30% of hospital-acquired pneumonia and is among the top three Gram-negative bacteria in hospital bacteremia. Infections are most severe in ICU patients, the immunocompromised, and those with invasive devices. Its biofilm formation on medical surfaces enables persistence and outbreaks (Chen et al., 2022).

The combination of strong antimicrobial resistance (AMR) and multiple virulence factors allows *K. pneumoniae* to cause severe, persistent, and hard-to-treat infections. Resistance arises from mechanisms such as extended-spectrum *β*-lactamases (ESBLs), carbapenemases, efflux pumps, and porin loss, which reduce treatment options (Wyres and Holt, 2018; Paczosa and Mecsas, 2016; Elsayed et al., 2022). Carbapenemase- and ESBL-producing strains are major global threats due to their ability to hydrolyze many β-lactam antibiotics. Key resistance genes include *bla*_KPC_, *bla*_OXA-48_, and *bla*_NDM_ for carbapenem resistance, and *bla*_CTX-M_ and *bla*_SHV_ for ESBL-mediated cephalosporin resistance (Ahmadi et al., 2025; Kebriaee et al., 2025). These plasmid-borne genes spread easily among isolates, driving multidrug resistance. The co-occurrence of carbapenemase and ESBL genes in single strains worsens resistance and complicates therapy. Monitoring their distribution is essential for surveillance and antimicrobial stewardship (Behzadi et al., 2020; Karampatakis et al., 2023; Halimi and Siroosi, 2025). Historically, *bla*_TEM_ was among the first ESBL genes identified, but its prevalence is now much lower than *bla*_CTX-M_ and *bla*_SHV_ in most *K. pneumoniae* strains (Edelstein et al., 2003; Peirano and Pitout, 2019).

Key virulence genes in *K. pneumoniae* include *rmpA*, which increases capsule production and causes a hypermucoviscous phenotype that resists phagocytosis (Russo and Marr, 2019; Shon et al., 2013). *iucA* supports aerobactin-mediated iron uptake, and *iroB* aids salmochelin synthesis for iron acquisition and immune evasion (Lam M. M. C. et al., 2018; Lam M. M. et al., 2018; Russo et al., 2014). *uge* contributes to lipopolysaccharide and capsule formation, enhancing host survival (Regué et al., 2004). *fimH1* mediates epithelial attachment and urinary tract colonization (Huang et al., 2022). While *mrkD* promotes biofilm formation, it is widespread in both classical and hypervirulent strains and offers limited diagnostic value (Alcántar-Curiel et al., 2013; Struve and Krogfelt, 2004). *wabG*, a glycosyltransferase for LPS biosynthesis, is essential for colonization and virulence (Izquierdo et al., 2003). The coexistence of virulence and AMR genes on mobile elements produces a dual-threat phenotype linked to higher mortality and nosocomial outbreaks (Wyres et al., 2020; Russo and Marr, 2019; Shon et al., 2013).

The *K. pneumoniae* infections are classified as pulmonary or extra-pulmonary. Pulmonary cases, including ventilator- and hospital-acquired pneumonia, mainly affect patients with chronic lung disease and show high mortality (30–50%) despite treatment (Chen et al., 2022). Extra-pulmonary infections include bacteremia, urinary tract infections, liver abscesses, meningitis, and soft tissue infections. It is a major cause of catheter-associated UTIs due to biofilm formation (Clegg and Murphy, 2016; Hafiz et al., 2023). Pulmonary forms are more common in adults with hospital exposure, while extra-pulmonary cases affect all ages. Infection site determines diagnosis, therapy, and outcome (Fostervold et al., 2024). Of note, *K. pneumoniae* comprises two main pathotypes: classical and hypervirulent. Classical strains cause opportunistic, drug-resistant infections in hospitalized or immunocompromised patients (Dong et al., 2022). Hypervirulent strains infect healthy individuals, causing severe diseases like liver abscesses, meningitis, and metastatic infections (Choby et al., 2020). Comparing these infection types is key for guiding therapy, infection control, and One Health–based public health measures. This study integrates clinical and microbiological data to improve outcomes, enhance surveillance, and reduce *K. pneumoniae*–related disease burden. It compares pulmonary and extra-pulmonary infections, focusing on differences between classical (cKp) and hypervirulent (hvKp) pathotypes, analyzing demographics, comorbidities, resistance profiles, and virulence factors to support targeted diagnosis and treatment.

## Methods

### Study design and sample collection

The present cross-sectional study included patients of all age groups, adults, children, and the elderly, who were suspected of having pulmonary or extra-pulmonary infections caused by *K. pneumoniae* (Supplementary Figure S1). Clinical assessment by qualified specialists included the following pulmonary and systemic signs: fever, tachypnea, productive cough, hypoxia, cyanosis, wheezes, hypotension, tachycardia, anemia, and pallor. A total of 600 clinical specimens were collected, preferably prior to antibiotic administration, from various hospitals across Egypt between March 2024 and January 2025. All samples were collected following clinical assessment before antibiotic initiation. Patients already receiving antibiotics were excluded based on clinical records and physician confirmation. In emergency cases, sampling occurred prior to the first antibiotic dose, as verified through treatment logs. For pulmonary cases, expectorated sputum samples (*n* = 100) were obtained from patients with suspected *K. pneumoniae* pneumonia. Sputum samples were included only when they met acceptable quality criteria based on microscopic examination (≤10 squamous epithelial cells and ≥25 polymorphonuclear leukocytes per low-power field), following Bartlett’s grading system, to minimize contamination from oropharyngeal flora. For extra-pulmonary infections, 500 specimens were collected, including urine, blood, cerebrospinal fluid (CSF), synovial fluid, and aspirated pus or fluid (100 samples of each type) from patients suspected of extra-pulmonary *K. pneumoniae* infections. Each sample was first inoculated into enrichment nutrient broth and incubated at 37 °C for 18–24 h under aerobic conditions. A loopful of the enriched culture was then streaked onto MacConkey agar for selective isolation of Gram-negative bacteria. Samples yielding at least 10^6^ uniform colonies suspected to be *K. pneumoniae* on MacConkey agar were considered positive.

### Identification of the recovered isolates

Identification to the species level was carried out following standard microbiological procedures. Initial screening included Gram staining to determine cell morphology, followed by culture on MacConkey and Eosin Methylene Blue agar (Oxoid, England). Presumptive *K. pneumoniae* isolates were further confirmed using conventional biochemical tests as described by Quinn et al. (2011). Automated identification was performed with the VITEK-2 system (bioMérieux, France) using the ID-GNB card for Gram-negative bacteria, following the manufacturer’s instructions, and results were interpreted automatically using version 2.01 software. Additional confirmation was achieved with the API 20E identification system (bioMérieux, Marcy l’Etoile, France). *Escherichia coli* ATCC 25922 and *K. pneumoniae* ATCC 700603 were included as a quality control strain throughout the identification process. All confirmed isolates were stored at −80 °C in Luria–Bertani (LB) broth supplemented with 15% (v/v) glycerol for long-term preservation.

### Characterization of classical and hypervirulence *Klebsiella pneumoniae*

#### Phenotypic characterization

##### String test

It is a simple phenotypic assay used to identify the hypermucoviscosity phenotype in *K. pneumoniae*, often linked to hypervirulent strains. A sterile inoculating loop is touched to a single colony grown on an agar plate (usually for 18–24 h) and then lifted slowly; formation of a viscous “string” measuring more than 5 mm between the loop and colony is considered a positive result, indicating overproduction of capsular polysaccharide. This test is rapid, inexpensive, and frequently used in clinical and research settings for preliminary screening of hypermucoviscous isolates (Shon et al., 2013).

##### Serum killing assay

It was performed to assess the ability of *K. pneumoniae* isolates to resist complement-mediated killing; a phenotype often associated with hypervirulent strains. Briefly, fresh pooled normal human serum (NHS) was obtained from healthy donors with no history of recent infection and filtered through a 0.22 μm membrane to remove debris. Mid-log phase bacterial cultures were washed and adjusted to approximately 1 × 10^6^ CFU/mL in phosphate-buffered saline (PBS). Equal volumes of bacterial suspension and NHS were mixed and incubated at 37 °C with gentle agitation. Viable counts were determined at 0, 1, 2, and 3 h by plating serial dilutions on nutrient agar, and survival rates were calculated as the percentage of CFU at each time point relative to time zero. Isolates were classified into serum sensitivity phenotypes (highly sensitive, intermediately sensitive, or resistant) according to previously described criteria. Heat-inactivated serum (56 °C for 30 min) served as a negative control to confirm complement-mediated effects (Podschun and Ullmann, 1998; Russo et al., 2011).

##### Chrome Azurol S (CAS) assay

Siderophore activity was determined through the Chrome Azurol S (CAS) technique. Each *K. pneumoniae* strain was cultivated in iron-deficient M9 minimal medium at 37 °C with continuous agitation for 24 h. Culture supernatants were obtained by centrifugation at 10,000 × g for 10 min and combined in equal volumes with freshly prepared CAS reagent according to Schwyn and Neilands (1987). After standing for 1 h at ambient temperature, absorbance readings were taken at 630 nm. Siderophore levels were expressed as a percentage reduction in absorbance relative to an uninoculated reference sample. To visually confirm production, isolates were additionally inoculated onto CAS agar and inspected for orange zones surrounding colonies, which denote iron-sequestering capability.

##### Serotyping by capsule swelling (Quellung reaction)

Capsular typing (K-serotyping) was carried out using the capsule swelling or Quellung method. Fresh colonies were emulsified in sterile saline on a clean microscope slide and mixed with a drop of type-specific antisera (Statens Serum Institute, Copenhagen, Denmark). A coverslip was placed on the mixture, and the preparation was examined under a phase-contrast microscope at 1000 × magnification. An isolate was considered positive when the capsule appeared enlarged and more refractile compared with the saline control. Each strain was tested sequentially against antisera targeting prevalent K types (K1, K2, K5, K20, K54, K57), following the previous described procedures (Wang et al., 2019; Feizabadi et al., 2013; Xu et al., 2024). *K. pneumoniae* ATCC 13883 (K2) and *K. pneumoniae* ATCC 43816 (K1) were used as positive controls; however, *Escherichia coli* ATCC 25922 was included as the negative control to verify assay specificity. These standard strains were kindly provided from the Animal Production Research Institute in Dokki, Giza, Egypt.

### Genotypic characterization of Hypervirulence genes

The presence of hypervirulence-associated genes (*rmpA*, *iucA*, and *iroB*) in *K. pneumoniae* isolates was determined using conventional PCR. Genomic DNA was extracted from overnight cultures using a commercial bacterial DNA extraction kit (Qiagen, Germany) following the manufacturer’s instructions. DNA concentration and purity were verified using a NanoDrop spectrophotometer, and working aliquots were prepared at approximately 50 ng/μL. Each PCR reaction (25 μL total volume) contained 12.5 μL of 2 × EmeraldAmp MAX PCR Master Mix (Takara, Japan), 0.5 μL of each forward and reverse primer (10 pmol/μL), 2 μL of template DNA, and nuclease-free water to make up the final volume. PCR amplification was performed in a thermal cycler (Bio-Rad, USA) using gene-specific cycling conditions and annealing temperatures as listed in Table 1. A well-characterized *K. pneumoniae* hypervirulent reference strain (positive control) and a no-template reaction (negative control) were included in each batch of amplification to ensure reliability. PCR products were separated by electrophoresis on a 1.5% (w/v) agarose gel prepared in 1 × TAE buffer, stained with ethidium bromide, and visualized under UV illumination using a gel documentation system (Bio-Rad, USA). Bands corresponding to the expected fragment sizes were recorded as positive results for the respective genes (Mai et al., 2023).

**Table 1:** Primer sequences, annealing conditions, and amplicon sizes for *K. pneumoniae* virulence genes.

*Gene*	Primer sequence (5′-3′)	Annealing Temp.	Amplicon size	References
*rmpA*	F: ACTGGGCTACCTCTGCTTCA R: CTTGCATGAGCCATCTTTCA	53 °C	535	Siu et al. (2011)
*uge*	F: TCTTCACGCCTTCCTTCACT R: GATCATCCGGTCTCCCTGTA	58 °C	534	Yu et al. (2006)
*fimH1*	F: GCCAACGTCTACGTTAACCTG R: ATATTTCACGGTGCCTGAAAA	43 °C	180	Wasfi et al. (2016)
*wabG*	F: ACCATCGGCCATTTGATAGA R: CGGACTGGCAGATCCATATC	58 °C	683	Yu et al. (2006)
*iucA*	F: AATCAATGGCTATTCCCGCTG R: CGCTTCACTTCTTTCACTGACAGG	59 °C	239	Russo et al. (2018)
*iroB*	F: CAAAAAAGCAGCAGAGGC R: TCACTGGCGGAATCCAACAC	59 °C	585	Russo et al. (2018)
*peg344*	F: CTTGAAACTATCCCTCCAGTC R: CCAGCGAAAGAATAACCCC	56 °C	508	Liao et al. (2020)

### Antimicrobial susceptibility testing

Bacterial identification and antimicrobial susceptibility testing were carried out using the VITEK-2 automated system (bioMérieux, Marcy l’Etoile, France). Initial identification of *K. pneumoniae* isolates was performed using the VITEK-2 ID-GNB card according to the manufacturer’s instructions. Confirmed isolates were then subjected to antimicrobial susceptibility testing using the VITEK-2 AST-GN04 card. Both procedures were conducted independently for each isolate using separate modules of the same instrument to ensure reproducibility.

In parallel, antimicrobial susceptibility was assessed using the Kirby–Bauer disk diffusion method on Mueller–Hinton agar and MIC determination with the VITEK-2 system against a panel of clinically relevant antimicrobials from different classes. The tested antibiotics included aminoglycosides (Amikacin, AMK), *β*-lactam/β-lactamase inhibitor combinations (Ampicillin/Sulbactam, SAM; Piperacillin, PIP), cephalosporins (Cefotaxime, CTX; Cefoxitin, FOX), fluoroquinolones (Ciprofloxacin, CIP; Levofloxacin, LVX), monobactams (Aztreonam, ATM), phenicols (Chloramphenicol, CHL), nitrofurans (Nitrofurantoin, NIF), tetracyclines (Doxycycline, DOX), carbapenems (Imipenem, IPM), and folate pathway inhibitors (Trimethoprim/Sulfamethoxazole, SXT). Interpretations of inhibition zones and minimum inhibitory concentration (MIC) values followed the Clinical and Laboratory Standards Institute (Clinical and Laboratory Standards Institute, 2024) guidelines. MICs were determined using the VITEK-2 system through kinetic fluorescence analysis, with results automatically processed by version 2.01 of the software after overnight incubation at 35 ± 2 °C. Of note, *K. pneumoniae* ATCC 700603 (ESBL-positive control) and *E. coli* ATCC 25922 (susceptible control) were used in accordance with CLSI recommendations. These standard strains were kindly provided from the Animal Production Research Institute in Dokki, Giza, Egypt In this study, multidrug resistance (MDR) was defined according to the criteria proposed by Magiorakos et al. (2012), as resistance to at least one antimicrobial agent in three or more distinct antimicrobial categories. This standardized definition was applied to classify the resistance profiles of all *K. pneumoniae* isolates in the present work.

### Phenotypic detection of resistance genes

#### Detection of metallo-β-lactamase (MBLs)

Metallo-β-lactamase production was detected using the combined disk synergy test (CDST) with 0.1 M ethylenediaminetetraacetic acid (EDTA). Briefly, two imipenem disks (Oxoid, Basingstoke, UK) were placed 30 mm apart (center-to-center) on an agar plate previously inoculated with the test strain. EDTA solution was applied to one of the disks, and the plates were incubated under standard conditions. The assay was performed in triplicate, and a result was considered positive when the inhibition zone diameter around the EDTA-containing disk was at least 7 mm greater than that of the disk without EDTA (Franklin et al., 2006).

#### Molecular characterization of resistance and other virulence genes

DNA was extracted using a commercial kit (Qiagen, Valencia, CA, USA) according to the manufacturer’s instructions. PCR amplification was performed in 25 μL reaction volumes containing 1 μL of bacterial DNA template, 12.5 μL of DreamTaq Green PCR Master Mix (2×) (Thermo Fisher Scientific), 1 μL of each primer, and nuclease-free water to bring the volume to 25 μL. Amplifications were carried out on a gradient PCR thermocycler (Analytik Jena, Germany). Cycling conditions for resistance genes, including carbapenemase-encoding genes (*bla*_KPC,_ *bla*_OXA-48_, *bla*_NDM_) and extended-spectrum β-lactamase (ESBL) gene (*bla*_CTX-M_), were optimized individually for each primer set as described in Table 2. Other virulence genes from *K. pneumoniae* isolates were amplified following previously described protocols (Table 1). Positive and negative controls were included in all runs, and PCR unidirectional workflow guidelines were strictly observed. *K. pneumoniae* ATCC 13883 (K2) and *K. pneumoniae* ATCC 43816 (K1) were used as positive controls for the PCR detection of virulence-associated genes (*rmpA*, *rmpA2*, *iucA*, *iroB*, *peg-344*). *Escherichia coli* ATCC 25922 served as the negative control. For the detection of resistance genes, *K. pneumoniae* ATCC 700603 (ESBL-positive control) and *E. coli* ATCC 25922 (susceptible control) were used in accordance with CLSI recommendations. These standard strains were kindly provided from the Animal Production Research Institute in Dokki, Giza, Egypt Amplified products were analyzed by electrophoresis on 2% agarose gels stained with ethidium bromide (0.5 μg/mL) at 70 V for 45 min, visualized under a UV transilluminator, and photo documented. Product sizes were estimated to use a 100 bp DNA ladder (Thermo Scientific, Molecular Biology).

**Table 2:** Primers used for detection of antimicrobial resistance genes, with sequences, annealing temperatures, amplicon sizes.

Gene	Primer sequence (5′-3′)	Tm	Amplicon size (bp)	References
*bla*_OXA-48_	GCTTGATCGCCCTCGATT GATTTGCTCCGTGGCCGAAA	60 °C	281	Azimi et al. (2014)
*bla*_KPC_	TGTTGCTGAAGGAGTTGGGC ACGACGGCATAGTCATTTGC	72 °C	340	Mlynarcik et al. (2016)
*bla*_NDM_	TAAAATACCTTGAGCGGGC AAATGGAAACTGGCGACC	72 °C	439	Mlynarcik et al. (2016)
*bla*_CTX-M_	AAAAATGATTCGGCTCCAGAA TGCCAGATTCGCTCTCAAAG	54 °C	483	Namaei et al. (2017)

### Statistical analysis

Statistical analysis compared hvKp and cKp across phenotypic features, virulence markers, resistance profiles and serotypes. Continuous and categorical variables were summarized as frequencies. Univariable comparisons used chi-square testing for categorical variables. Logistic regression models were used to examine the independent association of specimen type, age group and resistance profiles with hvKp status. Variables were entered as binary predictors and hvKp versus cKp was used as the dependent variable. Multicollinearity and complete separation were monitored during model fitting. The significance level of *p-value* < 0.05 was used to determine statistical significance for all analyses.

Other statistical analyses were conducted in R (version 4.x). Continuous variables were examined for distributional assumptions and standardized (z-scores) before visualization. Pairwise associations between clinical, virulence, and antimicrobial resistance variables were assessed using Pearson’s correlation for approximately normally distributed data, with correlation strength quantified using the correlation coefficient (*r-value*). Both positive (*r-value* > 0) and negative (*r-value* < 0) correlations were evaluated, with the magnitude of *r* interpreted as weak (*r-value* < 0.3), moderate (0.3 ≤ *r-value* < 0.7), or strong (*r-value* ≥ 0.7). Negative correlations were interpreted as inverse relationships between features (e.g., increased resistance associated with decreased presence of a virulence marker). Correlation matrices were generated with stats: cor and Hmisc (for *p* values), and both correlation coefficients and significance levels were visualized using ggcorrplot or corrplot. Heat maps of standardized data, with clinical, virulence, and antimicrobial resistance variables represented as presence/absence (coded as 0/1), were produced using pheatmap or Complex Heatmap, applying Euclidean distance and complete linkage for hierarchical clustering of both rows (isolates) and columns (features). In some analyses, distances based on 1 – Spearman’s *ρ* were also evaluated, with Spearman’s rank correlation coefficient (ρ) reported alongside *r* for non-normally distributed data. Missing values were imputed using the feature-wise median for continuous data or mode for binary data, and analyses were repeated as sensitivity checks. Plot annotations, such as hvKp vs. cKp classification and specimen type, were added as side bars, and color scales were centered at 0 to highlight relative enrichment.

## Results

### Phenotypic and genotypic profiling of pulmonary and extra-pulmonary *Klebsiella pneumoniae* isolates

The *K. pneumoniae* was identified in 103 cases among 600 clinical specimens analyzed, 43 from pulmonary and 60 from extra-pulmonary sources, representing an overall prevalence of 17.2%. Of note, identification results obtained using the API 20E system were fully consistent with those from the VITEK-2 ID-GNB card, showing 100% concordance across all 103 *K. pneumoniae* isolates, with no discrepancies detected. *K. pneumoniae* accounted for 43% of pulmonary disease cases, with 43 isolates recovered from 100 cases. In contrast, its overall prevalence among extra-pulmonary cases was 12% (60 out of 500). Therefore, Pulmonary samples, specifically sputum, showed a notably higher isolation rate compared to extra-pulmonary samples. *K. pneumoniae* was recovered from multiple body sites at varying frequencies, with the urinary tract being the most common among extra-pulmonary cases. Of note, blood cultures were positive in 16% of cases (16/100), urine samples showed a 19% yield (19/100), cerebrospinal fluid (CSF) had an 11% detection rate (11/100), synovial fluid yielded 8% (8/100), and aspirated fluid from necrotizing fasciitis lesions revealed 6% (6/100). Regarding gender distribution, a total of 63 isolates were recovered from male patients and 40 from female patients, indicating a slightly higher incidence of *K. pneumoniae* infection among males. Interestingly, the graphical abstract provided a concise visual summary of the main findings and their relationships in this study (Supplementary Figure S2).

**Figure 1:**
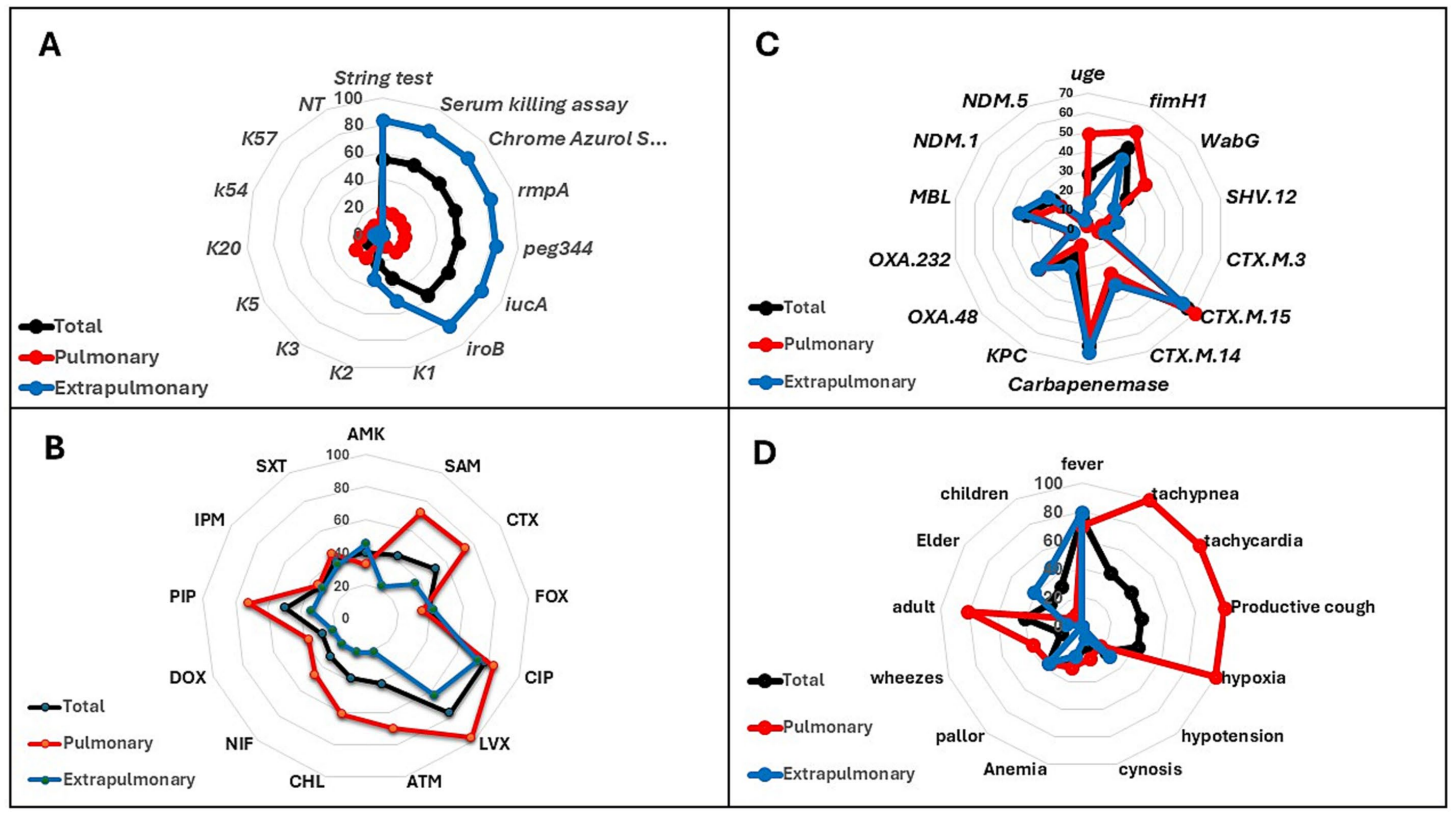
Radar chart analysis of *K. pneumoniae* isolates stratified by infection site (pulmonary vs. extrapulmonary). **(A)** Distribution of virulence-associated determinants, including phenotypic assays (string test, serum killing, chrome azurol S siderophore assay) and key virulence genes (*rmpA, peg344, iucA, iroB*) and capsular serotypes (K1, K2, K3, K5, K20, K54, K57). **(B)** Antimicrobial susceptibility profiles to commonly tested antibiotics: AMK, SAM, CTX, FOX, CIP, LVX, ATM, CHL, NIF, DOX, PIP, IPM, and SXT. **(C)** Prevalence of resistance genes, carbapenemase determinants (*bla*_CTX-M_*, bla*_OXA-48_*, bla*_NDM_*, bla*_KPC_) and other virulence genes (*uge, fimH1, wabG*). **(D)** Clinical and demographic associations, including patient age group (children, adults, elderly) and major clinical manifestations (fever, tachypnea, tachycardia, productive cough, hypoxia, hypotension, cyanosis, anemia, pallor, wheezes). Black line = total isolates; red line = pulmonary isolates; blue line = extrapulmonary isolates.

Approximately 83.3% (50 out of 60) of the extra-pulmonary isolates showed hypermucoviscosity in the string test and higher activity in the serum killing and Chrome Azurol S assays. These isolates also harbored the *rmpA, peg-344, iucA,* and *iroB* genes (Supplementary Figure S3), suggesting enhanced serum resistance and siderophore production; therefore, they were identified as hvKp (Table 3, Supplementary Table S1, Figure 1A). These isolates were also more frequently associated with K1 and K2 capsular types. In contrast, 83.7% (36 out of 43) of the pulmonary isolates exhibited opposite phenotypic and genotypic characteristics; therefore, they were classified as cKp (Table 3, Figure 1A). Among pulmonary isolates, the most common serotypes were K5 (23.3%) and K3 (20.9%), followed by K20 (16.3%), while K1 (9.3%) and K2 (4.7%) were less represented. In contrast, extra-pulmonary isolates were dominated by K1 (50%) and K2 (33.4%), whereas other serotypes were infrequent (K5, 5%; K20, 6.7%; K54, 3.4%; K57, 1.7%). No extra-pulmonary isolates were classified as K3 or NT (Figure 1A).

**Table 3:** Distribution of classical and hypervirulent *K. pneumoniae* (cKp and hvKp) isolates from pulmonary and extra-pulmonary infections.

	Pulmonary isolates	Extra-pulmonary isolates	Total
cKp	36	10	46
hvKp	7	50	57
Total	43	60	103

### Differential phenotypic and genotypic characterizations of hypervirulent versus classical *Klebsiella pneumoniae* isolates

**Figure 2:**
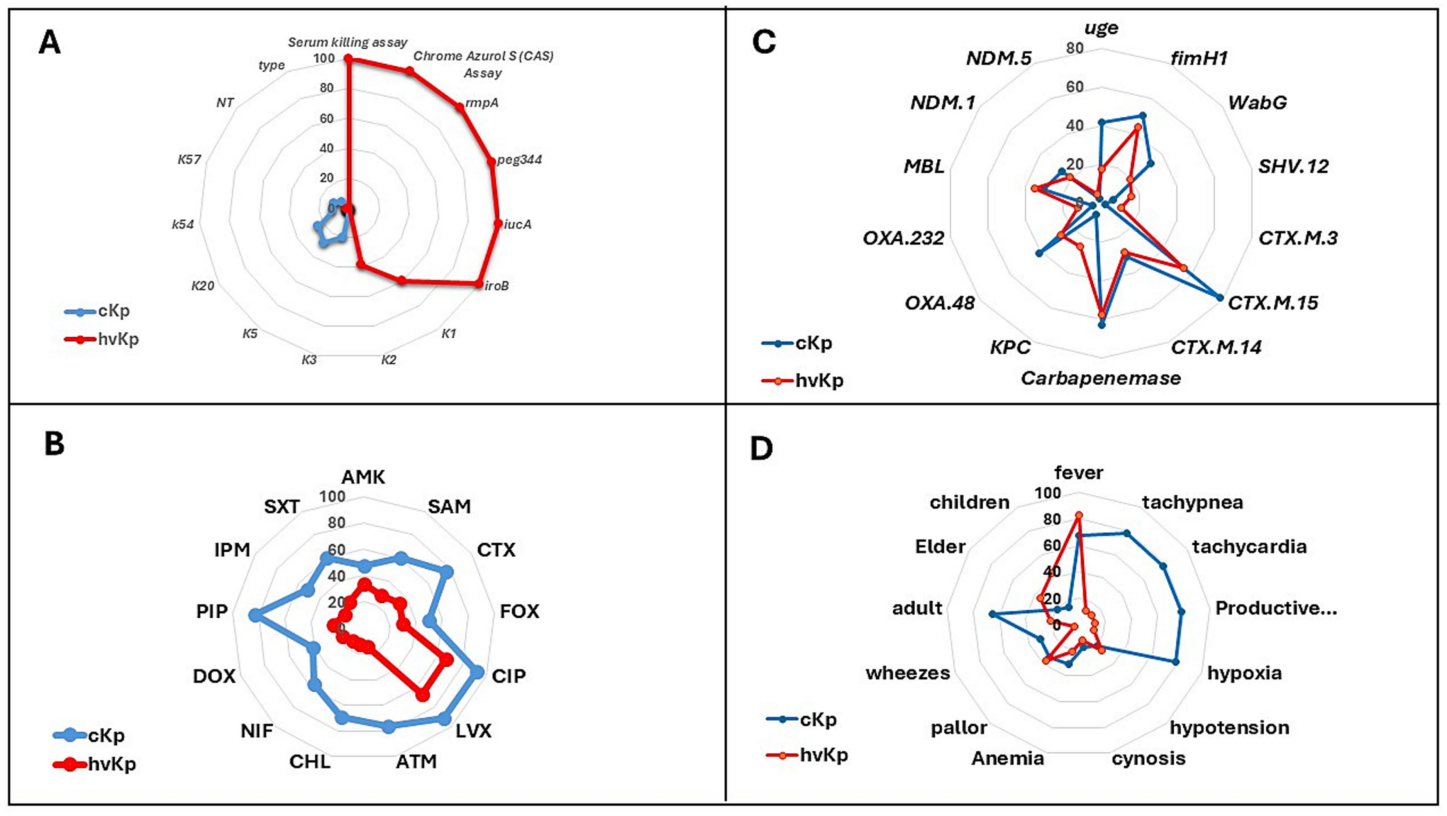
Comparative profiling of classical (*cKp*) and hypervirulent (*hvKp*) *K. pneumoniae* isolates. **(A)** Distribution of virulence determinants including phenotypic assays (string test, serum killing, chrome azurol S siderophore assay) and major virulence genes (*rmpA, peg344, iucA, iroB*) with capsular serotypes (K1, K2, K3, K5, K20, K54, K57, NT). **(B)** Antimicrobial susceptibility patterns to commonly tested antibiotics: AMK, SAM, CTX, FOX, CIP, LVX, ATM, CHL, NIF, DOX, PIP, IPM, and SXT, showing higher resistance in cKp compared to hvKp. **(C)** Prevalence of resistance genes, carbapenemase determinants (*bla*_CTX-M_*, bla*_OXA-48_*, bla*_NDM_*, bla*_KPC_) and other virulence genes (*uge, fimH1, wabG*). **(D)** Clinical and demographic associations, including patient age group (children, adults, elderly) and major clinical manifestations (fever, tachypnea, tachycardia, productive cough, hypoxia, hypotension, cyanosis, anemia, pallor, wheezes). Blue lines represent classical *K. pneumoniae* (cKp); red lines represent hypervirulent *K. pneumoniae* (hvKp).

**Figure 3:**
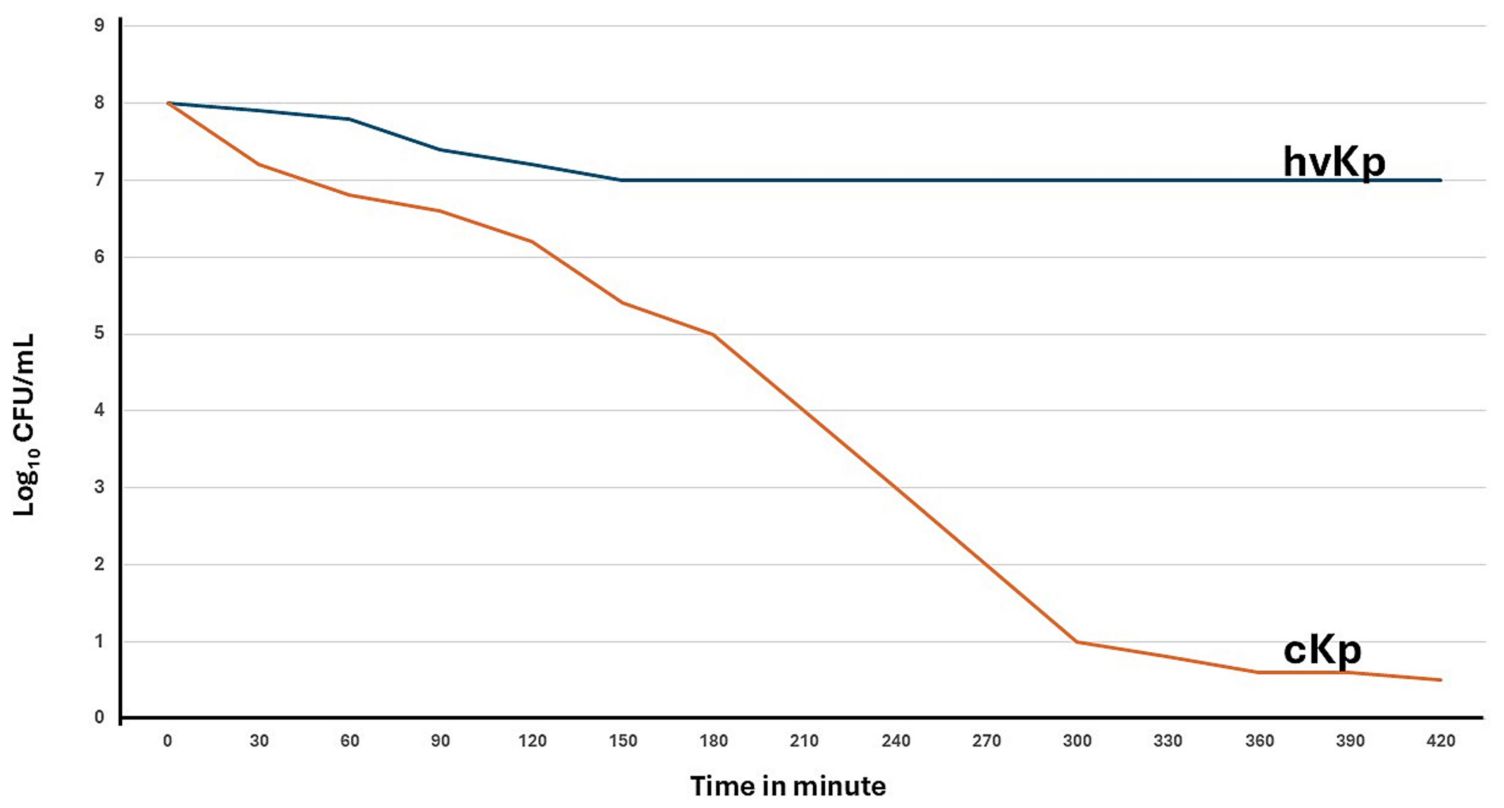
Serum killing assay of classical and hypervirulent *K. pneumoniae* isolates. The survival of *K. pneumoniae* strains was evaluated following exposure to normal human serum over time. The hypervirulent strain (hvKp) maintained stable viability, showing complete resistance to serum-mediated killing throughout the 420-min assay. In contrast, the classical strain (cKp) exhibited a rapid and progressive decline in viable counts, reaching complete elimination by the end of the experiment. These results demonstrate the enhanced serum resistance characteristic of hvKp compared to cKp. The X-axis shows time in minutes across the full duration of the experiment, and the Y-axis shows bacterial concentration measured as log₁₀ CFU per milliliter.

Out of 103 *K. pneumoniae* isolates, 55.3% (57/103) were phenotypically and genotypically identified as hvKp and 44.7% (46/103) as cKp. The hvKp strains tested positive in the string test (Supplementary Figure S4), Chrome Azurol S (CAS) assay, and serum killing assay, whereas the cKp strains were negative in these tests (Table 3, Figure 2A). The serum killing assay showed that cKp was rapidly eliminated over time, while hvKp remained resistant and maintained stable viability throughout the assay period (Figure 3). Molecular analysis confirmed hvKp in 57 of 103 isolates (55.3%) based on the presence of the *rmpA*, *iucA, peg-344 and iroB* genes. (Figure 2A; Table 4). The strong concordance between phenotypic methods and genotypic detection of these markers supports the classification of these isolates as true hvKp. In contrast, 46 of 103 isolates (44.7%) tested negative in all phenotypic assays and lacked these virulence-associated genes and were therefore identified as cKp (Table 5). Our results confirmed that there was no absolute association between strain type and clinical site, as both hvKp and cKp were recovered from pulmonary and extra-pulmonary infections. Among the 43 pulmonary isolates, 16.3% (7/43) were phenotypically identified as hvKp, while the remaining 83.7% (36/43) were cKp. In contrast, of the 60 extra-pulmonary isolates, 83.3% (50/60) were hvKp and 16.7% (10/60) were cKp (Table 3, Figure 1A). Notably, all hvKp isolates corresponded exclusively to K1 and K2 serotypes, whereas the remaining serotypes were detected only among cKp (Figure 2A).

**Table 4:** Antimicrobial resistance, virulence, and resistance gene characteristics of hypervirulence *K. pneumoniae* isolates.

Isolates	Antimicrobial resistance profile	Resistance gene profiling	Virulence genes profiling
hvKp1	SAM, LVX		*rmpA, peg344, peg344, iucA, iroB, uge, fimH1*
hvKp2	SAM, LVX	*bla*_CTX-M_	*rmpA, peg344, iucA, iroB, wabG*
hvKp3	LVX	*bla*_CTX-M_	*rmpA, peg344, iucA, iroB, uge, fimH1*
hvKp4	SAM, CTX, CIP, LVX	*bla*_CTX-M,_ *bla*_OXA-48_	*rmpA, peg344, iucA, iroB, wabG*
hvKp5	CIP, LVX	*bla*_OXA--48_ *, bla*_NDM_	*rmpA, peg344, iucA, iroB, uge, fimH1, WabG*
hvKp6	CIP, LVX	*bla*_KPC_	*rmpA, peg344, iucA, iroB, fimH1*
hvKp7	CIP, LVX, DOX	*bla*_KPC_	*rmpA, peg344, iucA, iroB*
hvKp8	AMK, SAM, FOX, LVX	*bla*_CTX-M_ *, bla*_NDM_	*rmpA, peg344, iucA, iroB, uge, fimH1*
hvKp9	AMK, CTX, FOX, NIF, IPM	*bla*_OXA-48_ *, bla*_NDM_	*rmpA, peg344, iucA, iroB, uge, fimH1*
hvKp10	FOX, LVX		*rmpA, peg344, iucA, iroB, WabG*
hvKp11	AMK, SAM, CTX, ATM	*bla*_CTX-M_ *bla*_NDM_	*rmpA, peg344, iucA, iroB, WabG*
hvKp12	SAM, SXT	*bla*_CTX-M_ *, bla*_NDM_	*rmpA, peg344, iucA, iroB, fimH1*
hvKp13	CTX, ATM, PIP	*bla*_CTX-M_ *, bla*_NDM_	*rmpA, peg344, iucA, iroB, wabG*
hvKp14	AMK, ATM	*bla*_CTX-M_	*rmpA, peg344, iucA, iroB, wabG*
hvKp15	AMK, CTX, CIP, LVX, DOX, IPM, SXT	*bla*_OXA-48_	*rmpA, peg344, iucA, iroB, wabG*
hvKp16	AMK, SAM, FOX, CIP, LVX, ATM, CHL, NIF, DOX, IPM, SXT	*bla*_CTX-M_	*rmpA, peg344, iucA, iroB, wabG*
hvKp17	AMK, CTX, CIP, LVX, CHL, PIP, IPM, SXT	*bla*_CTX-M,_ *bla*_KPC_	*rmpA, peg344, iucA, iroB*
hvKp18	AMK, CIP, ATM	*bla*_CT-M_	*rmpA, peg344, iucA, iroB*
hvKp19	AMK, FOX	*bla*_CTX-M_ *, bla*_KPC_	*rmpA, peg344, iucA, iroB, uge, fimH1*
hvKp20	CTX, FOX, PIP	*bla*_CTX-M_ *, bla*_KPC_	*rmpA, peg344, iucA, iroB, fimH1*
hvKp21	AMK, DOX	*bla*_CTX-M_	*rmpA, peg344, iucA, iroB, uge, fimH1*
hvKp22	FOX	*bla*_CTX-M_ *, bla*_KPC_	*rmpA, peg344, iucA, iroB, uge, fimH1*
hvKp23	AMK, SAM, CTX	*bla*_CTX-M_ *, bla*_OXA-48_ *, bla*_NDM_	*rmpA, peg344, iucA, iroB, uge, fimH1*
hvKp24	CTX, FOX, NIF	*bla*_CTX-M_ *, bla*_OXA-48_ *, bla*_NDM_	*rmpA, peg344, iucA, iroB*
hvKp25	AMK, SAM, CTX, FOX	*bla*_CTX-M_	*rmpA, peg344, iucA, iroB*
hvKp26	DOX		*rmpA, peg344, iucA, iroB*
hvKp27	SAM, CTX, CIP, LVX	*bla*_CTX-M_	*rmpA, peg344, iucA, iroB*
hvKp28	CTX, FOX, CIP, LVX	*bla*_CTX-M_	*rmpA, peg344, iucA, iroB*
hvKp29	AMK, SAM, CIP, LVX	*bla*_OXA-48_	*rmpA, peg344, iucA, iroB, fimH1*
hvKp30	AMK, CTX, FOX	*bla*_CTX-M,_ *bla*_NDM_	*rmpA, peg344, iucA, iroB, uge, fimH1*
hvKp31	CIP, ATM	*bla*_CTX-M_	*rmpA, peg344, iucA, iroB*
hvKp32	CIP, LVX, SXT	*bla*_OXA-48_	*rmpA, peg344, iucA, iroB, fimH1*
hvKp33	CTX, FOX, CIP, LVX	*bla*_OXA-48_	*rmpA, peg344, iucA, iroB, fimH1*
hvKp34	FOX, CIP, PIP	*bla*_CTX-M,_ *bla*_OXA-48_*, bla*_NDM_	*rmpA, peg344, iucA, iroB, fimH1*
hvKp35	CIP, LVX	*bla*_CTX-M,_ *bla*_OXA-48_*, bla*_NDM_	*rmpA, peg344, iucA, iroB, fimH1*
hvKp36	AMK, SAM, CIP, LVX	*bla*_OXA-48_	*rmpA, peg344, iucA, iroB, fimH1*
hvKp37	CIP	*bla*_KPC_	*rmpA, peg344, iucA, iroB*
hvKp38	CIP, LVX	*bla*_KPC_	*rmpA, peg344, iucA, iroB, fimH1*
hvKp39	CIP, PIP	*bla*_CTX-M_	*rmpA, peg344, iucA, iroB*
hvKp40	CTX, FOX, CIP, LVX	*bla*_KPC_	*rmpA, peg344, iucA, iroB, fimH1*
hvKp41	SAM, CIP, LVX, ATM, CHL, DOX, IPM, SXT	*bla*_KPC_	*rmpA, peg344, iucA, iroB*
hvKp42	CIP, LVX, PIP	*bla*_KPC_	*rmpA, peg344, iucA, iroB*
hvKp43	CIP, LVX, PIP	*bla*_KPC_	*rmpA, peg344, iucA, iroB*
hvKp44	CTX, CIP, LVX	*bla*_KPC_	*rmpA, peg344, iucA, iroB*
hvKp45	CIP, LVX, PIP	*bla*_KPC_ *, bla*_NDM_	*rmpA, peg344, iucA, iroB*
hvKp46	CIP, LVX, NIF		*rmpA, peg344, iucA, iroB*
hvKp47	SAM, CIP, LVX		*rmpA, peg344, iucA, iroB*
hvKp48	CIP, LVX, DOX, PIP, IPM, SXT	*bla*_CTX-M_ *, bla*_OXA-48_	*rmpA, peg344, iucA, iroB*
hvKp49	AMK, FOX, CIP, LVX, ATM, CHL, PIP, IPM, SXT	*bla*_OXA-48_	*rmpA, peg344, iucA, iroB*
hvKp50	SAM, CIP, LVX, NIF, DOX, PIP, IPM, SXT	*bla*_CTX-M_ *, bla*_OXA-48_	*rmpA, peg344, iucA, iroB, fimH1, wabG*
hvKp51	CIP, LVX, CHL, SXT	*bla*_CTX-M_	*rmpA, peg344, iucA, iroB*
hvKp52	AMK, CTX, FOX, CIP, LVX, NIF, DOX, PIP, IPM, SXT		*rmpA, peg344, iucA, iroB*
hvKp53	CTX, CIP, LVX		*rmpA, peg344, iucA, iroB, fimH1*
hvKp54	CIP, LVX, CHL	*bla*_CTX-M_	*rmpA, peg344, iucA, iroB, fimH1*
hvKp55	AMK, CTX, FOX, CIP, LVX, CHL, NIF, DOX, PIP, IPM, SXT	*bla*_OXA-48_ *, bla*_NDM_	*rmpA, peg344, iucA, iroB, fimH1, wabG*
hvKp56	CIP, LVX, SXT	*bla*_CTX-M_	*rmpA, peg344, iucA, iroB*
hvKp57	AMK, SAM, CIP, LVX	*bla*_CTX-M_	*rmpA, peg344, iucA, iroB*

The antimicrobial resistance profile lists antibiotics exhibiting phenotypic resistance, including Amikacin (AMK), Ampicillin–sulbactam (SAM), Cefotaxime (CTX), Cefoxitin (FOX), Ciprofloxacin (CIP), Levofloxacin (LVX), Aztreonam (ATM), Chloramphenicol (CHL), Nitrofurantoin (NIF), Doxycycline (DOX), Piperacillin (PIP), Imipenem (IPM), and Trimethoprim–sulfamethoxazole (SXT). The virulence gene profiling comprises major genes linked to capsule formation and adhesion such as *rmpA*, *peg344, iucA, iroB, uge, fimH1*, and *wabG*. The resistance gene profiling includes β-lactamase and carbapenemase determinants, notably *bla_CTX-M_* variants, *bla_KPC_, bla_OXA-48_* and *bla_NDM_* variants. The shaded isolates represented those that exhibited multidrug-resistant (MDR) patterns.

**Table 5:** Distribution of antimicrobial resistance, virulence, and resistance gene profiles among classical *K. pneumoniae.*

Isolates	Antimicrobial resistance profile	Resistance gene profiling	Virulence genes profiling
CKP1	SAM, CTX, CIP, LVX, ATM, CHL, PIP	*bla*_CTX-M_	*uge, fimH1*
CKP2	SAM, CTX, CIP, LVX, ATM, CHL, NIF, PIP, SXT	*bla*_CTX-M_	*WabG*
CKP3	SAM, CTX, FOX, CIP, LVX, ATM, CHL, NIF, DOX, PIP, IPM, SXT	*bla*_OXA-48_	*WabG*
CKP4	AMK, SAM, CTX, FOX, CIP, LVX, ATM, CHL, NIF, DOX, PIP, IPM, SXT	*bla*_OXA-48_	*WabG*
CKP5	AMK, SAM, CTX, LVX, ATM, CHL, DOX, PIP, IPM, SXT	*bla*_OXA-48_	*uge, fimH1, wabG*
CKP6	SAM, CTX, CIP, LVX, ATM, CHL, NIF, PIP	*bla*_OXA-48_	*WabG*
CKP7	SAM, CTX, CIP, LVX, ATM, CHL, NIF, PIP		*uge, fimH1, wabG*
CKP8	SAM, CTX, CIP, LVX, ATM, CHL, NIF, PIP	*bla*_CTX-M_	
CKP9	SAM, CTX, LVX, ATM, NIF, PIP	*bla*_CTX-M_	*fimH1*
CKP10	AMK, SAM, CTX, FOX, CIP, LVX, ATM, CHL, DOX, PIP, IPM, SXT	*bla*_CTX-M_	*uge, fimH1*
CKP11	CTX, CIP, LVX, ATM, CHL, PIP	*bla*_CTX-M_	*fimH1, wabG*
CKP12	SAM, CTX, CIP, LVX, ATM, CHL, NIF, PIP, SXT	*bla*_CTX-M_	*uge, fimH1*
CKP13	AMK, SAM, CTX, FOX, CIP, LVX, ATM, CHL, DOX, PIP, IPM, SXT	*bla*_CTX-M_ *, bla*_OXA-48_ *, bla*_NDM_	*uge, fimH1*
CKP14	SAM, CIP, LVX	*bla*_CTX-M_	*uge, fimH1, wabG*
CKP15	SAM, CTX, CIP, LVX, ATM, NIF, PIP	*bla*_CTX-M_ *, bla*_OXA-48_	*uge*
CKP16	AMK, CTX, FOX, CIP, LVX, ATM, CHL, NIF, DOX, PIP, IPM, SXT	*bla*_CTX-M_ *, bla*_OXA-48_	
CKP17	AMK, CTX, FOX, CIP, LVX, ATM, CHL, DOX, PIP, SXT	*bla*_CTX-M_	*uge, fimH1*
CKP18	SAM, CTX, CIP, LVX, ATM, CHL, NIF, PIP	*bla*_CTX-M_ *, bla*_OXA-48_	
CKP19	SAM, LVX	*bla*_CTX-M_ *, bla*_NDM_	*uge, fimH1*
CKP20	AMK, SAM, CTX, FOX, CIP, LVX, ATM, CHL, NIF, DOX, PIP, IPM, SXT	*bla*_CTX-M_ *, bla*_OXA-48_	*wabG*
CKP21	SAM, LVX	blaCTX-M, blaNDM	
CKP22	SAM, CTX, CIP, LVX, ATM, CHL, NIF, DOX, PIP, IPM, SXT	*bla*_CTX-M_ *, bla*_OXA-48_	*uge, fimH1*
CKP23	AMK, SAM, CTX, FOX, CIP, LVX, ATM, CHL, NIF, PIP, IPM, SXT	*bla*_CTX-M_	*fimH1, wabG*
CKP24	AMK, SAM, CTX, FOX, CIP, LVX, ATM, CHL, PIP, IPM, SXT	*bla*_CTX-M_ *, bla*_NDM_	*uge, fimH1*
CKP25	AMK, SAM, CTX, FOX, CIP, LVX, CHL, PIP, SXT	*bla*_CTX-M_	
CKP26	AMK, SAM, CTX, FOX, CIP, LVX, ATM, CHL, PIP, IPM, SXT	*bla*_CTX-M_	*uge*
CKP27	SAM, CTX, CIP, LVX, ATM, CHL, NIF, PIP	*bla*_CTX-M_ *, bla*_NDM_	*fimH1*
CKP28	AMK, SAM, CTX, FOX, CIP, LVX, ATM, CHL, NIF, DOX, PIP, IPM, SXT	*bla*_CTX-M_ *, bla*_NDM_	*uge, fimH1, wabG*
CKP29	AMK, SAM, CTX, FOX, CIP, LVX, ATM, CHL, DOX, PIP, IPM, SXT	*bla*_CTX-M_ *, bla*_NDM_	*fimH1*
CKP30	AMK, SAM, CTX, FOX, CIP, LVX, ATM, CHL, NIF, DOX, PIP, IPM, SXT		*fimH1*
CKP31	CIP, LVX, DOX	*bla*_CTX-M,_	uge
CKP32	CIP, LVX, DOX	*bla*_KPC_	*uge, fimH1, wabG*
CKP33	CTX, CIP, ATM, NIF, PIP	*bla*_CTX-M_ *, bla*_OXA-48_ *, bla*_NDM_	*uge*
CKP34	CTX, CIP, LVX, ATM, NIF, PIP	*bla*_KPC_	*uge, fimH1*
CKP35	CTX, CIP, LVX, ATM, NIF, DOX, PIP		*wabG*
CKP36	SAM, CTX, FOX, CIP, LVX, ATM, CHL, PIP, IPM, SXT	*bla*_CTX-M_ *, bla*_OXA-48_	
CKP37	AMK, FOX, CIP, LVX, CHL, NIF, PIP, IPM, SXT	*bla*_CTX-M_ *, bla*_NDM_	*wabG*
CKP38	AMK, FOX, CIP, LVX, ATM, DOX, PIP, IPM, SXT	*bla*_CTX-M_ *, bla*_NDM_	*wabG*
CKP39	AMK, CTX, FOX, CIP, LVX, ATM, CHL, DOX, PIP, IPM, SXT	*bla*_CTX-M_ *, bla*_KPC_	
CKP40	AMK, FOX, CIP, LVX, CHL, NIF, PIP, IPM, SXT	*bla*_CTX-M_ *, bla*_OXA-48_ *, bla*_NDM_	*fimH1*
CKP41	AMK, CTX, FOX, CIP, IPM, SXT	*bla*_OXA-48_	*fimH1*
CKP42	AMK, CTX, FOX, CIP, CHL, NIF, PIP, IPM, SXT	*bla*_CTX-M_	
CKP43	AMK, FOX, CIP, LVX, ATM, CHL, PIP, IPM, SXT	*bla*_CTX-M_ *, bla*_OXA-48_ *, bla*_NDM_	*fimH1*
CKP44	CIP, ATM, NIF, DOX, IPM, SXT	*bla*_CTX-M_ *, bla*_OXA-48_ *, bla*_NDM_	*uge*
CKP45	CIP, LVX, NIF, DOX, PIP	*bla*_CTX-M_ *, bla*_OXA-48_	
CKP46	AMK, CTX, FOX, CIP, LVX, ATM, CHL, NIF, IPM, SXT	*bla*_CTX-M_ *, bla*_OXA-48_	

The antimicrobial resistance profile lists antibiotics exhibiting phenotypic resistance, including Amikacin (AMK), Ampicillin–sulbactam (SAM), Cefotaxime (CTX), Cefoxitin (FOX), Ciprofloxacin (CIP), Levofloxacin (LVX), Aztreonam (ATM), Chloramphenicol (CHL), Nitrofurantoin (NIF), Doxycycline (DOX), Piperacillin (PIP), Imipenem (IPM), and Trimethoprim–sulfamethoxazole (SXT). The virulence gene profiling comprises major genes linked to capsule formation and adhesion such as *rmpA, peg344, iucA, iroB, uge, fimH1*, and wabG. The resistance gene profiling includes β-lactamase and carbapenemase determinants, notably *bla*_*CTX*-M_ variants, *bla_KPC_*, *bla_OXA-48_* and *bla_NDM_* variants. The shaded isolates represented those that exhibited non-multidrug-resistant (MDR) patterns.

### Antimicrobial resistance patterns

Among the 103 *K. pneumoniae* isolates, antimicrobial resistance was widely distributed across multiple antibiotic classes, with substantial variation between pulmonary and extra-pulmonary sources (Figure 1B), and between classical (cKp) and hypervirulent (hvKp) strains (Figure 2B; Tables 4, 5, Supplementary Table S1). Of note, antibiogram results obtained using Kirby–Bauer disk diffusion were fully consistent with those from the VITEK-2 system, showing 100% concordance across all 103 *K. pneumoniae* isolates, with no discrepancies detected. The highest resistance rates were observed for fluoroquinolones, with ciprofloxacin (CIP) and levofloxacin (LVX) each exhibiting resistance in 77.7% of isolates. Beta-lactam resistance was also pronounced, particularly for cefotaxime (CTX) at 52.5% and ampicillin-sulbactam (SAM) at 42.8%. In contrast, lower resistance rates were noted for doxycycline (DOX) (28.2%), nitrofurantoin (NIF) (32.1%), and imipenem (IPM) (33.1%), suggesting that some susceptibility to these agents was retained (Figure 1B).

### Pulmonary versus extra-pulmonary resistance profiles

When classified by infection site, pulmonary isolates demonstrated substantially higher resistance rates across multiple antimicrobial classes compared to extra-pulmonary isolates. Resistance to ampicillin–sulbactam (SAM; *β*-lactam/β-lactamase inhibitor combination) was recorded in 72.1% of pulmonary isolates, whereas only 21.7% of extra-pulmonary isolates were resistant. Similarly, resistance to cefotaxime (CTX; third-generation cephalosporin) was markedly higher in pulmonary isolates (74.4%) than in extra-pulmonary isolates (36.7%). Resistance within the fluoroquinolone class was elevated in both groups but reached particularly high levels for levofloxacin (LVX) in pulmonary isolates (97.7%) compared to 63.4% in extra-pulmonary cases. These trends mirrored the multidrug resistance (MDR) distribution, with pulmonary isolates showing an MDR rate of 72.1% versus 28.3% among extra-pulmonary isolates, indicating a significantly greater antimicrobial resistance burden in respiratory tract infections (Figure 1B).

### Comparative resistance between cKp and hvKp

Detailed resistance profiling revealed that classical *K. pneumoniae* (cKp) exhibited consistently higher resistance rates across nearly all antimicrobial agents compared to hypervirulent *K. pneumoniae* (hvKp). Resistance to cefotaxime (CTX; third-generation cephalosporin) was documented in 76.1% of cKp isolates versus 33.4% of hvKp isolates. Resistance to ciprofloxacin (CIP; fluoroquinolone) was 91.4% in cKp compared to 66.7% in hvKp, and resistance to aztreonam (ATM; monobactam) reached 76.1% in cKp but only 14.1% in hvKp. Similar trends were observed for chloramphenicol (CHL; phenicol antibiotic), with resistance in 69.6% of cKp versus 12.3% of hvKp, and for piperacillin (PIP; ureidopenicillin), with resistance in 82.7% of cKp compared to 22.9% of hvKp (Figure 2B).

### MDR prevalence and strain-type differences

The MDR analysis revealed a clear disparity between cKp and hvKp isolates. Multidrug resistance was detected in 36.8% (21/57) of hvKp isolates (Table 4) compared to 89.1% (41/46) of cKp isolates (Table 5). These findings indicated that while hvKp possessed enhanced virulence traits, it was generally more susceptible to antimicrobial agents than cKp. This inverse relationship between resistance and virulence carried important clinical implications, particularly for treatment strategies in severe infections. Not all MDR isolates carried a large number of resistance genes; some exhibited multidrug resistance despite harboring only a few, likely due to other phenotypic mechanisms unrelated to the investigated resistance genes.

### Resistance and virulence genes profiles

Generally, the phenotypic detection of *β*-lactam resistance was consistent with the genetic findings. The most prevalent ESBL determinant was *bla*_CTX-M_, detected in 65.1% of isolates (Figure 1C, Supplementary Figure S3). Pulmonary isolates carried more *bla*_CTX-M_ (69.8%) than extra-pulmonary ones (61.7%) (Figure 1C). Strain-type analysis revealed *bla*_CTX-M_ predominated in cKp (78.3%) compared to hvKp (54.4%), reflecting its stronger link to multidrug resistance. Carbapenemase genes were widespread, with *bla*_OXA-48_ (33.1%) being the most common, followed by *bla*_KPC_ (16.6%). Regarding Metallo-lactamase (MBL) genes, *bla*_NDM_ was present in 23.4% of the detected *K. pneumoniae* isolates. Extra-pulmonary isolates showed higher overall carbapenemase carriage, largely due to *bla*_KPC_ prevalence (21.7%), while pulmonary isolates more often carried *bla*_OXA-48_ (32.6%). Between strain types, cKp exhibited more *bla*_OXA-48_ (41.4%) and *bla*_NDM_ (26.1%), whereas hvKp carried more *bla*_KPC_ (24.6%) (Figure 2C).

Regarding the additional virulence-associated genes, *uge*, *fimH1*, and *wabG*, beyond the key hypervirulence markers (*rmpA*, *iucA*, and *iroB*), all *K. pneumoniae* isolates were analyzed for their distribution. The *fimH1* gene was the most prevalent (46.7%) and encodes the type 1 fimbrial adhesin responsible for host cell attachment. The *uge* (28.2%) and *wabG* (25.3%) genes followed in frequency and are involved in capsular and lipopolysaccharide biosynthesis. Pulmonary isolates had consistently higher detection rates of these genes compared to extra-pulmonary isolates, with *uge* present in 48.8% versus 13.4%, *fimH1* in 55.8% versus 40%, and *wabG* in 37.2% versus 16.7%, respectively (Figure 1C). When stratified by strain type, cKp isolates carried these virulence genes at rates relatively close to those of hvKp. In cKp, *fimH1* was found in 50%, compared to 43.9% in hvKp (Figure 2C). This indicates that although hvKp is characterized by distinct hypervirulence markers (such as *rmpA, iucA, iroB*), cKp shows a relatively similar prevalence of classical adhesion and membrane-related genes that may likewise contribute to colonization and persistence.

### Clinical presentation

Fever was the predominant symptom (75.8%), followed by productive cough, tachypnea, tachycardia, and hypoxia (41.8% each). Other signs included pallor (35%), anemia (25.3%), hypotension (24.3%), wheezes (15.6%), and cyanosis (14.6%). Patients spanned all age groups: adults 40.8%, children 31.1%, and elderly 28.1%. Pulmonary infections were marked by universal respiratory signs (100%) and occurred mainly in adults (81.4%); fever (69.8%), pallor (34.9%), wheezes (37.2%), and anemia (30.2%) were also common, while hypotension (18.6%) and cyanosis (23.3%) were less frequent (Figure 1D). Extra-pulmonary infections lacked respiratory signs, but fever (80%), pallor (35%), and anemia (21.7%) were common, with hypotension (28.4%) and cyanosis (8.4%) less frequent; these occurred mostly in elderly (41.7%) and pediatric patients (46.7%). cKp infections were strongly linked to systemic and respiratory signs (78.3%), fever (67.4%), wheezes (30.5%), and anemia (30.5%), and affected mainly adults (65.3%). In contrast, hvKp infections were more often associated with fever (82.5%) and minimal respiratory involvement (12.3%), with pallor (36.9%) and anemia (21.1%) present but wheezes rare (3.6%); these predominated in elderly (35.1%) and pediatric patients (43.9%) (Figure 2D).

### Cluster analysis of *Klebsiella pneumoniae* isolates based on antimicrobial resistance patterns

**Figure 4:**
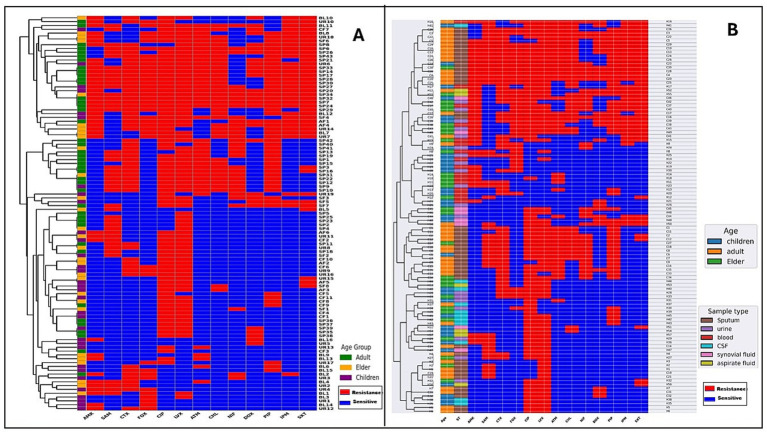
Hierarchical clustering of antimicrobial resistance profiles in *K. pneumoniae* isolates. **(A)** Clustering of isolates according to infection site (pulmonary vs. extra-pulmonary) and **(B)** clustering according to pathotypes (classical *K. pneumoniae* [cKp] vs. hypervirulent *K. pneumoniae* [hvKp]). The X-axis displays the panel of tested antimicrobials, each represented as a separate column showing isolate-level resistance or susceptibility patterns. Red indicates resistance and blue indicates susceptibility across tested antimicrobials: AMK (amikacin), SAM (ampicillin-sulbactam), CTX (cefotaxime), FOX (cefoxitin), CIP (ciprofloxacin), LVX (levofloxacin), ATM (aztreonam), CHL (chloramphenicol), NIF (nitrofurantoin), DOX (doxycycline), PIP (piperacillin), IPM (imipenem), and SXT (trimethoprim-sulfamethoxazole). Color keys: age group (blue = children, orange = adults, green = elderly) and sample type (pink = sputum, brown = urine, red = blood, gray = cerebrospinal fluid, purple = synovial fluid, yellow = aspirate fluid).

As observed in Figure 4A, pulmonary isolates exhibited a markedly higher burden of resistance determinants, with most strains classified as multidrug-resistant (MDR). In contrast, extrapulmonary isolates were generally more susceptible to antimicrobial agents, and only a minority were MDR. In the dendrogram, pulmonary isolates are grouped together in distinct MDR-rich clusters, characterized by a predominance of red squares, indicating resistance across multiple drug classes. Extrapulmonary isolates, by contrast, tended to form separate susceptibility-rich clusters enriched in blue squares, highlighting a strong segregation of AMR profiles by anatomical site. While this separation was generally robust, a few cross-group placements were observed: some extrapulmonary strains were embedded within MDR pulmonary clusters, and conversely, a small number of pulmonary isolates were positioned within susceptible extrapulmonary clusters.

When analyzed by pathotype (Figure 4B), a distinct divergence in resistance profiles was evident. The cKp strains displayed a higher prevalence of multidrug resistance, clustering together in MDR-dense branches enriched in red squares. The hvKp strains, in contrast, formed predominantly susceptibility-rich clusters, with blue squares dominating, supporting the notion that hypervirulence and high-level drug resistance are generally inversely correlated. Similar to the pulmonary versus extrapulmonary pattern, a small number of intergroup placements were noted: hvKp strains positioned within MDR-dense cKp clusters, and occasional cKp strains embedded within hvKp-dominated susceptible clusters. These exceptions suggest that although hvKp typically remain drug-susceptible and cKp are often MDR, the boundaries are not absolute. Horizontal gene transfer or convergent adaptation could facilitate the emergence of hvKp strains with acquired resistance or cKp strains with attenuated resistance profiles.

### Hierarchical clustering of *Klebsiella pneumoniae* isolates based on phenotypic and genotypic detection of key virulence factors and capsular types, and resistance genes

**Figure 5:**
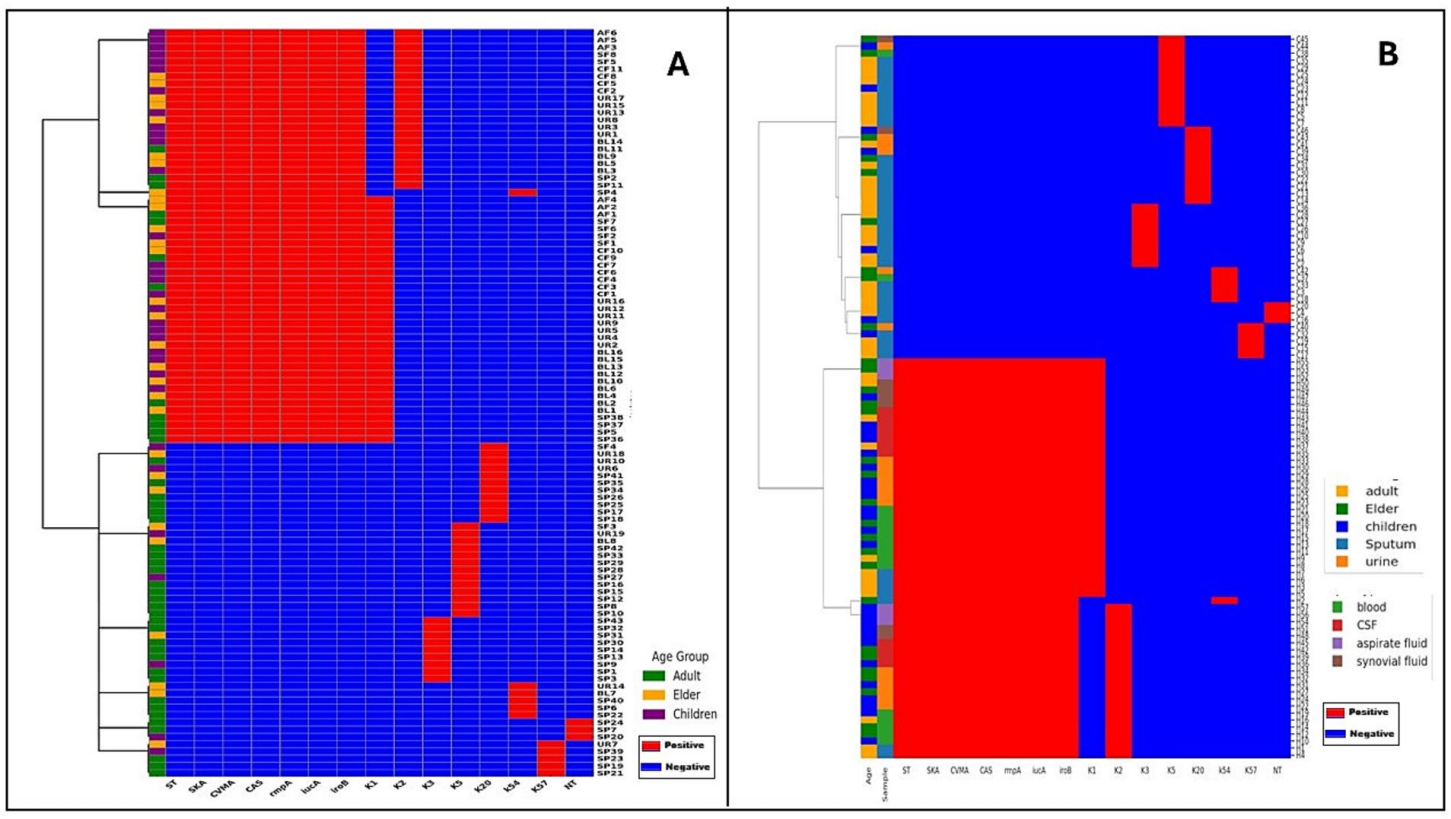
Hierarchical clustering of the existence of key virulence markers in *K. pneumoniae* isolates using both phenotypic and genotypic methods. **(A)** Clustering of isolates according to infection site (pulmonary vs. extra-pulmonary) and **(B)** clustering according to pathotypes (classical *K. pneumoniae* [cKp] vs. hypervirulent *K. pneumoniae* [hvKp]). The X-axis shows the set of virulence genes and serotype markers tested in the isolates, with each column representing one marker and indicating its presence or absence. Red indicates presence and blue indicates absence of virulence genes and phenotypic traits, including ST (string test), SKA (serum killing assay), CAS (chrome azurol S assay), *rmpA*, *iucA*, *iroB*, and capsular serotypes K1, K2, K3, K5, K20, K54, K57, and NT (non-typeable). Color keys: age group (blue = children, orange = adults, green = elderly) and sample type (pink = sputum, brown = urine, red = blood, gray = cerebrospinal fluid [CSF], purple = synovial fluid, yellow = aspirate fluid).

**Figure 6:**
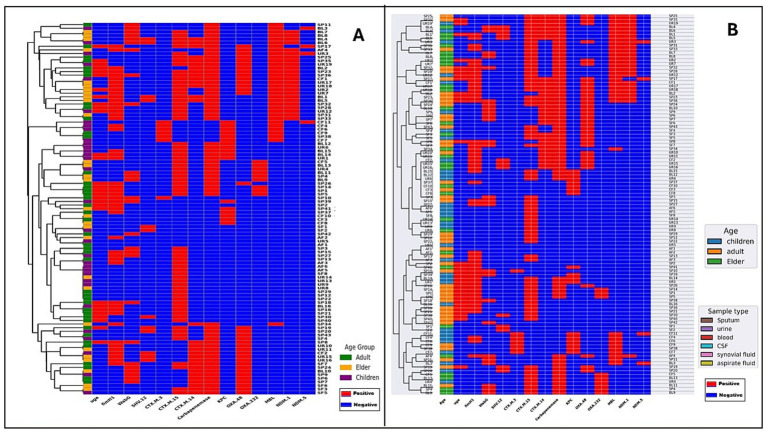
Heat map showing antimicrobial resistance (AMR) and other virulence genes in *K. pneumoniae* isolates. **(A)** Clustering of isolates according to infection site (pulmonary vs. extra-pulmonary) and **(B)** clustering according to pathotypes (classical *K. pneumoniae* [cKp] vs. hypervirulent *K. pneumoniae* [hvKp]). The X-axis displays the panel of resistance genes tested in the isolates, with each column representing one gene and indicating its presence or absence. Red indicates presence and blue indicates absence of resistance genes, carbapenemase determinants (*bla*_CTX-M_*, bla*_OXA-48_*, bla*_NDM_*, bla*_KPC_) and other virulence genes (*uge, fimH1, wabG*). Color keys: age group (blue = children, orange = adults, green = elderly) and sample type (purple = sputum, brown = urine, red = blood, gray = cerebrospinal fluid [CSF], pink = synovial fluid, yellow = aspirate fluid). The virulence genes shown in this figure are those other than the genetic markers used to distinguish hvKp from cKp. These genes were not used to identify hypervirulent *K. pneumoniae* but are found in both classical and hypervirulent strains.

Clustering analyses from key virulence-associated traits and capsular types (Figure 5) and resistance gene profiles in addition to non-specific virulence genes (Figure 6) revealed consistent patterns linking infection site and pathotype with antimicrobial resistance and virulence potential. In Figure 5A, pulmonary isolates clustered predominantly in phenotype-negative groups, showing weak or negative results in all phenotypic virulence assays and lacking key virulence-associated genes. Extrapulmonary isolates, in contrast, were frequently grouped in phenotype-rich clusters characterized by strong positive assay results, indicative of capsule production, biofilm formation, and iron acquisition—traits associated with increased invasive potential. In Figure 5B, hvKp strains harbored this key virulence genes enriched in capsular serotypes K1 and K2, as well as carrying virulence genes such as *rmpA*, *iucA*, and *iroB*. cKp strains, on the other hand, were grouped in low-virulence clusters, consistent with negative phenotypic assay profiles and the absence or low prevalence of these genetic markers. Some pulmonary isolates were clustered together with extra-pulmonary isolates, and conversely, certain extra-pulmonary isolates were grouped more closely with the pulmonary set. This overlap suggests that the genetic profiles of isolates are not strictly determined by their site of origin, and that virulence traits may be shared across both pulmonary and extra-pulmonary infections, reflecting a relative fluidity in their distribution.

In Figure 6A, pulmonary isolates clustered in resistance gene–rich branches, showing frequent carriage of *β*-lactamase genes (*bla*_CTX-M,_ *bla*_OXA-48_) and carbapenemase determinants. Extrapulmonary isolates generally formed separate clusters with fewer resistance genes and a lower prevalence of multidrug resistance determinants. In Figure 6B, cKp strains clustered in resistance gene–dense branches, often harboring multiple β-lactamase and carbapenemase genes, along with other non–key virulence genes. hvKp strains were largely located in clusters with sparse resistance gene content, reflecting their generally susceptible phenotype. A small number of hvKp isolates with extensive resistance genes appeared within cKp-dominated clusters, underscoring the potential emergence of strains combining hypervirulence and multidrug resistance.

### Dendrogram analysis based on clinical manifestations in relation to infection site and pathotype

**Figure 7:**
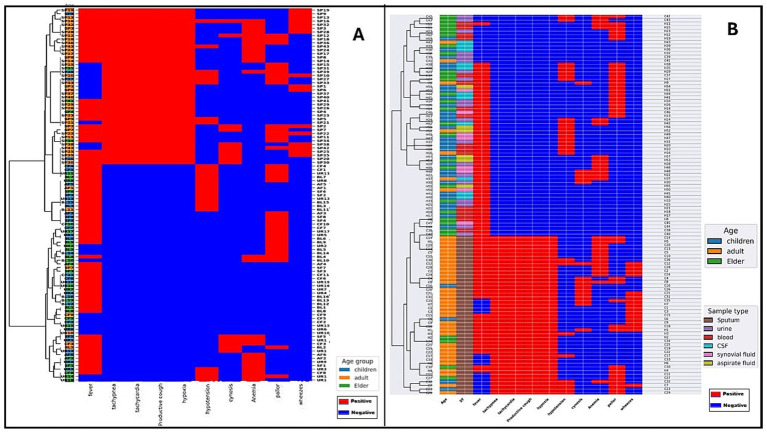
Dendrogram of *K. pneumoniae* isolates showing clinical symptom clustering by source and pathotype. **(A)** Clustering of isolates according to infection site, showing associated clinical symptoms such as fever, tachypnea, tachycardia, productive cough, hypoxia, hypotension, cyanosis, anemia, pallor, wheezes. **(B)** Clustering of isolates according to pathotypes, illustrating variation in clinical features across different infection sources. The X-axis shows the panel of clinical manifestations assessed in each case, with each column representing one symptom and indicating its presence or absence. Colored side bars indicate age groups (blue = children, orange = adults, green = elders), and sample types (purple = sputum, brown = urine, red = blood, cyan = CSF, pink = synovial fluid, yellow = aspirate fluid).

Clustering of clinical manifestations (Figure 7) revealed distinct symptom groupings that align with both infection site (Figure 7A) and pathotype (Figure 7B). Pulmonary isolates clustered predominantly with cases presenting the classic respiratory profile of fever, productive cough, hypoxia, and tachypnea. These symptom combinations formed a major branch of the dendrogram, strongly suggesting their association with respiratory infection. In contrast, extrapulmonary isolates were linked to a broader and more varied symptom distribution, including hypotension, anemia, and other systemic signs such as tachycardia. These manifestations were dispersed across clusters, reflecting the diverse clinical presentations of invasive or disseminated infections.

When grouped by pathotype, cKp strains aligned closely with respiratory-associated clusters, again characterized by fever, productive cough, hypoxia, and tachypnea. hvKp strains showed a broader spectrum of presentations, with clusters containing severe systemic features such as hypotension, anemia, and tachycardia, consistent with their propensity to cause invasive extrapulmonary disease. The integration of clinical symptom clustering with microbiological classification indicates that symptom profiles are not random but are linked to isolate type. Pulmonary/cKp clusters tend to present with localized respiratory signs, whereas extrapulmonary/hvKp clusters are associated with more severe systemic manifestations. These findings support the hypothesis that pathogen-specific traits, rather than patient age or sample type alone, play a significant role in shaping clinical presentation, and that hvKp infections may carry a higher risk for severe systemic dissemination.

### Correlation analysis

#### Virulence, resistance, and serotype associations among hvKp and MDR isolates

The hvKp isolates were associated with a tight cluster of virulence genes and specific serotypes rather than resistance traits. The *rmpA*, *iucA*, *peg344*, and *iroB* consistently indicated hypervirulence. Other virulence genes (*uge*, *fimH1*, *wabG*) were conserved and did not differentiate hvKp from cKp. Most antimicrobials and resistance genes, including *bla*_CTX-M_, *bla*_KPC_, *bla*_OXA-48_, and *bla*_NDM_, showed no independent association with hvKp, except aztreonam (*p* < 0.05). hvKp clustered in K1, K2, and K57 serotypes, while cKp were dispersed with many nontypeable strains, and serotyping served as a strong categorical classifier. Regarding to the MDR fitness, it was linked to broad resistance across multiple drugs. Aztreonam, imipenem, chloramphenicol, piperacillin, trimethoprim–sulfamethoxazole, cefotaxime, nitrofurantoin, amikacin, ampicillin–sulbactam, doxycycline, cefoxitin, and ciprofloxacin were significant (*p* < 0.05). MDR isolates shared some hvKp virulence genes (*rmpA*, *iucA*, *iroB*, *peg344*), but background genes (*wabG*, *fimH1*, *uge*) were unrelated. Carbapenemase genes (*bla*_KPC_, *bla*_OXA-48_) drove MDR, while *bla*_CTX-M_ showed a weak correlation and *bla*_NDM_ was not associated. MDR reflects resistance mechanisms rather than hypervirulence.

#### Pearson’s correlations between infection sites, pathotype, resistance and clinical features

**Figure 8:**
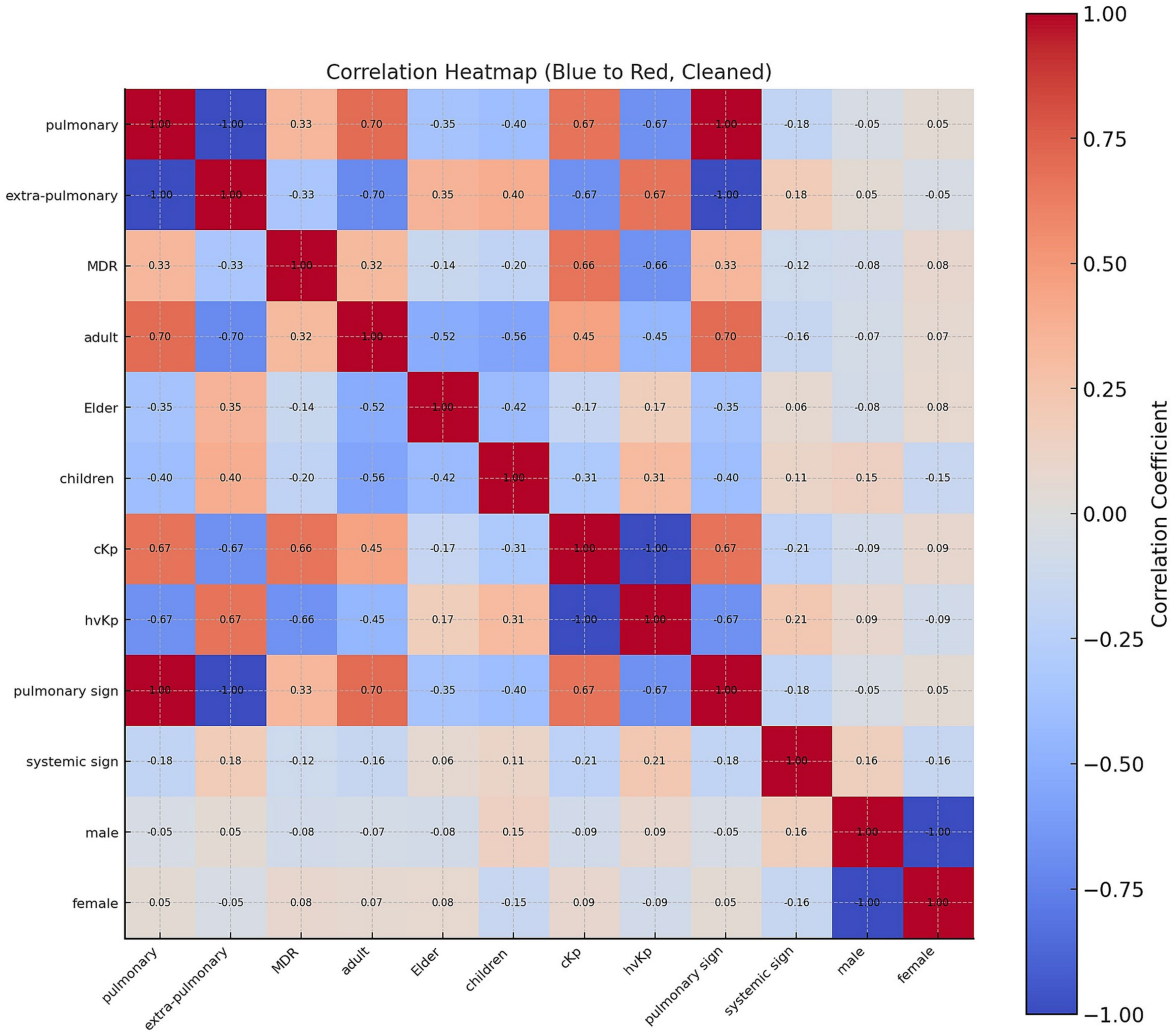
Correlation heatmap of clinical and bacterial variables in *K. pneumoniae* infections. The heatmap displays correlation coefficients among clinical features, demographic groups, and bacterial pathotypes. The X-axis displays the epidemiological, clinical and microbiological variables included in the correlation matrix, and the Y-axis lists the same set of variables in the same order to show their pairwise correlations. Red shading indicates positive correlations, while blue represents negative correlations, with intensity reflecting the strength of association. Variables included were infection site (pulmonary, extra-pulmonary), multidrug resistance (MDR), patient age groups (children, adult, elder), pathotypes (classical *K. pneumoniae* [cKp] and hypervirulent *K. pneumoniae* [hvKp]), and clinical manifestations (pulmonary signs, systemic signs).

Pearson’s correlation analysis revealed clear and biologically consistent associations between infection site, pathotype, antimicrobial resistance, age group, and clinical manifestations (Figure 8). Pulmonary infection was perfectly and inversely correlated with extrapulmonary infection (*r-value* = −1.00), reflecting their mutually exclusive classification. Pulmonary isolates were moderately associated with multidrug resistance (MDR) (*r-value* = 0.33) and strongly correlated with the cKp pathotype (*r-value* = 0.67), while being strongly negatively correlated with the hvKp pathotype (*r-value* = −0.67). Extrapulmonary isolates demonstrated the reverse pattern, correlating positively with hvKp (*r-value* = 0.67) and negatively with cKp (*r-value* = −0.67). MDR was strongly associated with cKp (*r-value* = 0.66) and inversely with hvKp (*r-value* = −0.66).

Age associations indicated that pulmonary infections were more frequent in adults (*r-value* = 0.70) and inversely related to both elderly (*r-value* = −0.35) and pediatric (*r-value* = −0.40) cases. Extrapulmonary infections correlated positively with elderly (*r-value* = 0.35) and pediatric patients (*r-value* = 0.40). Adult age showed moderate positive correlation with cKp (*r-value* = 0.45) and negative correlation with hvKp (*r-value* = −0.45), whereas pediatric cases correlated positively with hvKp (*r-value* = 0.29) and negatively with cKp (*r-value* = −0.29). Clinical signs followed similar trends: the presence of pulmonary signs correlated positively with pulmonary infection (*r-value* = 1.00) and cKp (*r-value* = 0.67), and negatively with extrapulmonary infection (*r-value* = −1.00) and hvKp (*r-value* = −0.67). Systemic signs showed weaker associations, with a mild positive correlation with extrapulmonary infection (*r-value* = 0.18) and hvKp (*r-value* = 0.18), and mild negative correlation with pulmonary infection (*r-value* = −0.18) and cKp (*r-value* = −0.18).

The correlation analysis showed weak associations between gender and other studied variables. Both male and female variables demonstrated very low correlation coefficients (r values between −0.16 and 0.09) with clinical and microbiological parameters such as infection site (pulmonary, extra-pulmonary), age group (adult, elder, children), and strain type (cKp, hvKp, MDR). These results suggest that gender had no significant correlation with the distribution or characteristics of *K. pneumoniae* isolates in this dataset (Figure 8).

Overall, these correlations reinforce the phenotypic and genotypic clustering results: Pulmonary/cKp infections tend to occur in adults, show higher MDR prevalence, and present predominantly with localized respiratory symptoms. Extrapulmonary/hvKp infections are more common in pediatric and elderly patients, are largely susceptible to antimicrobials, and are associated with systemic manifestations. This integrated pattern suggests that pathogen-specific traits, rather than patient demographics alone, play a decisive role in determining both the resistance profile and the clinical presentation.

## Discussion

The global rise in antimicrobial resistance poses a serious public health threat, intensifying the challenge of treating infections caused by opportunistic and multidrug-resistant pathogens (Mosallam et al., 2023; Bendary et al., 2022). The increasing prevalence of *K. pneumoniae*, especially hypervirulent (hvKp) infections, reflects its strong adaptive capacity and ability to colonize diverse anatomical sites, attributed to its virulence fitness. This strain shows increased virulence through capsule polysaccharide and siderophore overproduction, enabling resistance to neutrophil phagocytosis and complement attack (Hetta et al., 2025). Once limited to East Asia, these strains are now reported globally (Choby et al., 2020; Russo and Marr, 2019; Hetta et al., 2025). Their recent acquisition of antimicrobial resistance genes has created a high-risk combination of virulence and drug resistance, complicating detection and treatment (Choby et al., 2020). Multiple phenotypic and genotypic methods are used to differentiate hypervirulent hvKp from cKp. Phenotypically, hvKp shows a hypermucoviscous phenotype detectable by the string test, where a filament exceeding 5 mm forms when a colony is lifted with a loop (Zhu et al., 2021). hvKp colonies are larger, shiny, and sticky due to plasmid-regulated overproduction of capsular polysaccharides (Russo and Marr, 2019; Russo et al., 2024; Nguyen et al., 2024). Serum killing assays demonstrate hvKp resistance to complement-mediated lysis, unlike the serum-sensitive cKp (Al-Busaidi et al., 2024). The Chrome Azurol S (CAS) assay reveals higher siderophore production in hvKp, mainly aerobactin and salmochelin, whereas cKp primarily produces enterobactin. Serotyping through the Quellung reaction associates hvKp with K1 and K2 serotypes, while cKp exhibits diverse, less virulent capsule types (Shon et al., 2013). Genotypically, hvKp is identified by large plasmids such as pLVPK or pK2044, containing critical virulence genes such as *rmpA*, *rmpA2*, *iucA*, *iroB*, *peg-344*, and *wabG*, often plasmid-borne (Russo and Marr, 2019). These markers are widely recognized as reliable molecular indicators for distinguishing hvKp from cKp in clinical and epidemiological investigations (Dong et al., 2022; Lam M. M. C. et al., 2018; Harada and Doi, 2018).

The observed distribution in this study confirmed its role as a versatile pathogen capable of infecting various body systems. Urinary tract and pulmonary infections were the most frequent, consistent with global epidemiological patterns, highlighting the need for continuous surveillance amid rising antimicrobial resistance (Podschun and Ullmann, 1998). These results emphasize the importance of early and accurate diagnosis and strict infection control measures to limit healthcare-associated transmission. The detection of *K. pneumoniae* in bloodstream, cerebrospinal, synovial, and soft-tissue infections demonstrated its invasive nature and ability to cause severe, life-threatening diseases. With the global emergence of multidrug-resistant and hypervirulent strains, alternative therapeutic strategies targeting virulence mechanisms are essential to reduce dependence on traditional antibiotics (Ghaly et al., 2021; Abd El-Hamid et al., 2023; Ghaly et al., 2023). The infection patterns observed further underline the need for continuous monitoring, strong antimicrobial stewardship, and region-specific epidemiological research to guide empirical treatment and minimize disease burden (Gupta et al., 2011; Abdelrahman et al., 2024).

This study identified the capsular types K1 (33.1%) and K2 (21.4%) as the predominant serotypes, with a strong association to extra-pulmonary infections (50 and 33.4%, respectively). These findings supported previous evidence that K1 and K2 were the hallmark serotypes of hypervirulent *K. pneumoniae* (hvKp), often linked to invasive diseases such as liver abscesses and bacteremia (Shon et al., 2013; Russo and Marr, 2019). In contrast, pulmonary infections were more frequently associated with K3 (20.9%), K5 (23.3%), and K20 (16.3%), suggesting these stereotypes might have played a greater role in respiratory tract infections (Lin et al., 2012). Notably, all hvKp isolates in this study belonged exclusively to K1 and K2, while the other serotypes were restricted to classical *K. pneumoniae* (cKp). This phenotypic–genotypic–serotypic concordance highlighted the diagnostic value of combining capsular typing with molecular assays for hvKp detection (Paczosa and Mecsas, 2016; Struve et al., 2015). Given emerging reports of carbapenem-resistant hvKp (CR-hvKp), the predominance of K1 and K2 in systemic infections underscored the urgent need for routine surveillance and antimicrobial stewardship to mitigate the threat of multidrug-resistant hypervirulent strains (Lam M. M. C. et al., 2018; Lam M. M. et al., 2018; Zhang F. et al., 2016; Zhang Y. et al., 2016). Consistent with our findings, the hvKp strains generally exhibit stronger biofilm formation than classical strains. This enhanced biofilm capacity is driven by multiple inter-linked factors: over-expression of capsule-regulating genes such as *rmpA* and *rmpA2*, leading to increased polysaccharide production which enhances adherence and protects cells within the biofilm matrix (Russo and Marr, 2019). The hvKp commonly carry adhesin genes like *fimH1* and *mrkD*, which promote initial surface attachment, and increased siderophore genes (e.g., *iucA*, *iroB*) that enhance iron acquisition in nutrient-limited biofilm environments (Li and Ni, 2023). Furthermore, studies show that certain capsular types associated with hvKp (e.g., K1) form robust air-liquid interface biofilms (“floating” biofilms), which may contribute to persistence and resistance *in vivo* (Cubero et al., 2019). Together, these traits create a synergistic effect where virulence-associated factors originally linked to hypermucoviscosity and invasiveness also enhance biofilm formation, making hvKp more resilient and persistent than classical *K. pneumoniae* lineages.

The widespread antimicrobial resistance observed among *K. pneumoniae* isolates in this study highlighted the pathogen’s adaptability and clinical threat, particularly in pulmonary infections where resistance rates were markedly higher. Elevated resistance to fluoroquinolones (77.7%) and third-generation cephalosporins such as cefotaxime (52.5%) was consistent with global reports documenting rising resistance in respiratory isolates of *K. pneumoniae* (Paterson and Bonomo, 2005; Pitout and Laupland, 2008). In contrast, the retained susceptibility to imipenem and doxycycline in some isolates suggested potential therapeutic value for these agents, although carbapenem resistance continued to emerge worldwide (Martin and Bachman, 2018). The significantly higher multidrug resistance (MDR) burden in pulmonary isolates (72.1%) compared to extra-pulmonary isolates (28.3%) underscored the difficulty of treating respiratory tract infections, reflecting trends observed in international surveillance studies (Della Rocca et al., 2022). The combined findings also underscored two major clinical challenges: first, pulmonary infections were more frequently caused by highly resistant classical *K. pneumoniae* (cKp) strains, complicating empirical therapy; second, extra-pulmonary infections, although more often associated with hypervirulent *K. pneumoniae* (hvKp) and generally more susceptible to antibiotics, carried a greater risk of invasive disease and severe clinical outcomes. This disparity mirrored prior evidence, with cKp demonstrating higher resistance rates while hvKp remained more susceptible yet exhibited greater invasive potential (Shon et al., 2013; Paczosa and Mecsas, 2016). The inverse relationship between resistance and virulence reinforced the complexity of therapeutic strategies: while cKp posed challenges due to its high MDR prevalence (89.1%), hvKp represented a distinct clinical threat by causing severe systemic infections despite lower resistance levels. Together, these findings emphasized the urgent need for rapid strain-type identification and antimicrobial susceptibility testing to guide targeted treatment. Furthermore, continuous surveillance, antimicrobial stewardship, and region-specific epidemiological monitoring were essential to contain the dual threat posed by MDR-cKp and hvKp strains (Russo and Marr, 2019; Lam M. M. C. et al., 2018; Lam M. M. et al., 2018).

Clustering analysis revealed that pulmonary and extra-pulmonary isolates generally formed separate groups, reflecting their site-specific patterns of antimicrobial resistance, virulence genes, and clinical features (Russo and Marr, 2019; Muraya et al., 2022). However, some pulmonary isolates were located within extra-pulmonary clusters, and conversely, some extra-pulmonary isolates were grouped with pulmonary clusters. This overlap indicates that while pulmonary strains typically cause localized lung infection, they can disseminate and cause systemic or metastatic disease, a phenomenon well-documented in invasive *K. pneumoniae* infections such as bacteremia and urinary diseases (Choby et al., 2020; Shon et al., 2013). The presence of pulmonary isolates in extra-pulmonary clusters was supported by their similar resistance and virulence profiles, as well as shared clinical signs with systemic infections, suggesting that these strains maintain the fitness to cross anatomical barriers. Likewise, some extra-pulmonary isolates appearing in pulmonary clusters may reflect infection spreading into the lung through the bloodstream, or shared genetic traits that let them adapt to different body sites (Doorduijn et al., 2016; Russo et al., 2014). A similar trend was observed between cKp and hvKp: although cKp generally formed multidrug-resistant clusters and hvKp formed hypervirulent ones, some hvKp isolates clustered with cKp and vice versa. This intermixing reflects the growing recognition that *K. pneumoniae* can acquire both virulence and resistance traits, leading to hybrid lineages that make the difference between the two pathotypes less clear (Russo and Marr, 2019; Muraya et al., 2022; Mendes et al., 2023). Overall, these overlaps demonstrate that clustering trends are clear but not absolute, and they highlight the genetic adaptability of *K. pneumoniae*, which enables both pulmonary-to-systemic spread and the convergence of virulence with antimicrobial resistance. (Chen et al., 2022; Mendes et al., 2023).

The correlation patterns observed in this study are aligned with established distinctions between classical and hypervirulent *K. pneumoniae*. Pulmonary isolates were more closely linked with cKp and multidrug resistance, reflecting the well-known role of cKp as a leading cause of hospital-acquired pneumonia and ventilator-associated infections, where antimicrobial pressure selects for resistant strains (Russo and Marr, 2019; Muraya et al., 2022). In contrast, extra-pulmonary isolates showed stronger association with hvKp, consistent with its recognized capacity for hematogenous spread and metastatic complications, including urinary diseases, liver abscesses and endophthalmitis (Shon et al., 2013; Choby et al., 2020). These patterns suggest that the type of strain—especially whether it carries hypervirulence genes or resistance genes—plays a major role in deciding where the infection occurs and how it appears in patients, more than the host factors alone. Age-related associations also reflected epidemiological trends. Adult patients were more frequently affected by cKp-related pulmonary infections, likely due to higher healthcare exposure and comorbidities (Chen and Chen, 2021). Conversely, hvKp-associated extra-pulmonary infections were more common in pediatric and elderly populations, consistent with reports that hvKp can cause severe disease even in healthy children and older adults with diminished immunity (Han et al., 2024; Du et al., 2022).

Clinical correlations further underscored these differences: pulmonary infections presented predominantly with localized respiratory symptoms, while systemic signs such as anemia, hypotension, jaundice, septic shock, or altered mental status were more often linked to hvKp-dominated extra-pulmonary disease (Doorduijn et al., 2016). Together, these links show that *K. pneumoniae* falls along a spectrum, where both resistance and virulence factors shape where infections occur and how severe they become. This makes it important to track genetic, phenotypic, and clinical features together for early detection and better management. Finally, these combined genetic, phenotypic, and clinical analyses reflect the current understanding of *K. pneumoniae* biology: hvKp usually carries a clear set of virulence traits that allow it to infect many body sites, while cKp is more often linked to hospital settings and multidrug resistance. However, the overlaps and convergence we observed show that these strains lie on a spectrum rather than in strict categories. This highlights the importance of cluster-based multi-factor surveillance to guide targeted treatment and effective infection control.

## Conclusion

From a practical perspective, these findings emphasize the need for early recognition of hypervirulent and multidrug-resistant *K. pneumoniae* in routine clinical practice. Since pulmonary infections are more often caused by highly resistant cKp strains, clinicians should be cautious when selecting empiric therapy for hospital-acquired and ventilator-associated pneumonia, prioritizing rapid antimicrobial susceptibility testing to avoid treatment failure. Conversely, extra-pulmonary infections, particularly bloodstream or liver abscesses linked to hvKp (K1/K2), require prompt identification, as these strains often remain antibiotic-susceptible but can cause rapid and severe systemic disease. For infection control, the demonstrated overlap between pulmonary and extra-pulmonary clusters highlights the potential for cross-site spread, reinforcing the need for strict hospital hygiene, isolation protocols, and contact precautions to limit nosocomial transmission. Capsular typing and molecular assays should be integrated into diagnostic workflows where feasible, as they allow early detection of hvKp and help guide targeted therapy. At the public health level, the coexistence and convergence of resistance and virulence traits underscore the urgency of continuous surveillance, antimicrobial stewardship programs, and region-specific monitoring to inform empirical therapy. Together, these measures are essential to reduce morbidity, mortality, and the burden of *K. pneumoniae* in healthcare settings.

### Strengths and limitations

This study’s major strength lies in its comprehensive, multi-site sampling strategy and its integrated approach combining phenotypic, genotypic, and clinical data correlation. This design allowed a broader understanding of *K. pneumoniae* diversity across different infection sites while linking laboratory findings with patient outcomes. The inclusion of both classical and hypervirulent strains and the analysis of their antimicrobial resistance and virulence profiles provided a robust framework for clinical interpretation.

However, studying has certain limitations. It was conducted within a single-country scope, which may limit the generalizability of the findings to other regions with differing epidemiological trends. The molecular analysis was restricted to a selected gene panel, excluding some adhesion and resistance determinants such as *mrkD*. Additionally, whole-genome sequencing (WGS) was not performed, which would have enabled a more comprehensive exploration of genetic relationships, horizontal gene transfer, and emerging hybrid lineages. Future studies incorporating expanded gene targets and WGS across multiple geographic regions would provide deeper insights into the evolving epidemiology and pathogenic potential of *K. pneumoniae*. Further studies are needed to validate the clinical findings in a murine infection model using hvKp, cKp and hvKp–MDR hybrid isolates. The work will assess disease severity, bacterial load and survival, test antibiotic response and examine how MDR affects treatment failure. Gene knockout and complementation of *rmpA*, *iucA*, *iroB* and *peg344* will confirm their role in invasiveness, and cytokine profiling will characterize host responses. This approach will provide functional support for the clinical patterns and strengthen genotype–phenotype links.

## Data Availability

The original contributions presented in the study are included in the article/Supplementary material, further inquiries can be directed to the corresponding author.

## Supplementary material

SUPPLEMENTARY FIGURE S1 Flow chart summarizing the methodology for isolation, characterization, and analysis of *K. pneumoniae* isolates. A total of 600 clinical samples from pulmonary and extra-pulmonary infections were collected and processed using standard microbiological techniques. *K. pneumoniae* isolates were identified based on colony morphology, Gram staining, and biochemical testing. Phenotypic characterization included the string test for hypermucoviscosity, serum killing assay for serum resistance, Chrome Azurol S (CAS) assay for siderophore activity, and capsule serotyping by the Quellung reaction. Antimicrobial susceptibility was determined by the disk diffusion method according to CLSI guidelines, and multidrug resistance (MDR) was defined per Magiorakos et al. (2012). Molecular characterization involved PCR detection of virulence-associated genes (*rmpA, iucA, iroB, uge, fimH1,* and *wabG*). Statistical analysis integrated clinical, phenotypic, and genotypic data to differentiate classical (*cKp*) and hypervirulent (*hvKp*) isolates.

SUPPLEMENTARY FIGURE S2 Comparative analysis of pulmonary and extra-pulmonary K. pneumoniae isolates and their phenotypic, molecular, and resistance profiles. A total of 103 *K. pneumoniae* isolates were recovered, including 43 pulmonary and 60 extra-pulmonary isolates, with an overall prevalence of 17.2%. Among these, 46 isolates were classified as classical (*cKp*) and 57 as hypervirulent (*hvKp*). *cKp* isolates predominated in pulmonary infections, while *hvKp* isolates were mainly associated with extra-pulmonary infections. The majority of multidrug-resistant (MDR) strains (89.1%) belonged to *cKp*, particularly from pulmonary sources (72.1%), whereas *hvKp* isolates showed lower resistance (10.5%). Correlation analysis revealed that pulmonary/*cKp* infections were linked to adults and MDR phenotypes, while extra-pulmonary/*hvKp* infections occurred primarily in children and the elderly with systemic manifestations. Cluster analysis showed that pulmonary isolates formed MDR-dense clusters, while extra-pulmonary isolates grouped into susceptibility-rich clusters, underscoring the distinct clinical and resistance patterns between *cKp* and *hvKp*.

SUPPLEMENTARY FIGURE S3 Gel electrophoresis images showing PCR amplification of virulence and resistance genes among K. pneumoniae isolates. Panels **A–C** show the detection of virulence-associated genes used to differentiate hypervirulent (*hvKp*) from classical (*cKp*) strains, including *peg-344* (344 bp) (A), *iucA* (239 bp) **(B)**, and *iroB* (585 bp) **(C)**. Panels D–F display the amplification of antimicrobial resistance genes, with *bla*_OXA-48_ (763 bp) **(D)**, *bla*_KPC_ (340 bp) **(E)**, and multiplex PCR detection of, blaCTX-M gene **(F)**. Lane M represents the DNA molecular weight marker, lanes 2–19 correspond to individual K. pneumoniae isolates, lane 1 serves as the positive control, and lane 20 serves as the negative control. The presence of distinct bands confirms the amplification of specific virulence and resistance genes.

SUPPLEMENTARY FIGURE S4 Hypermucoviscosity assessment using the string test. A positive result is indicated by the formation of a viscous string measuring ≥5 mm when a bacterial colony is stretched upward using an inoculation loop from an agar plate, demonstrating the hypermucoviscous phenotype characteristic of hypervirulent *K. pneumoniae* (hvKp) isolates.
