# Supplementary figures and images for: Pulmonary and extra-pulmonary infections caused by classical and hypervirulent Klebsiella pneumoniae: a prospective cross-sectional study
Source: Front Microbiol. 2026 Jan 2;16:1707017. doi: 10.3389/fmicb.2025.1707017 (PMC12808397; doi:10.3389/fmicb.2025.1707017)

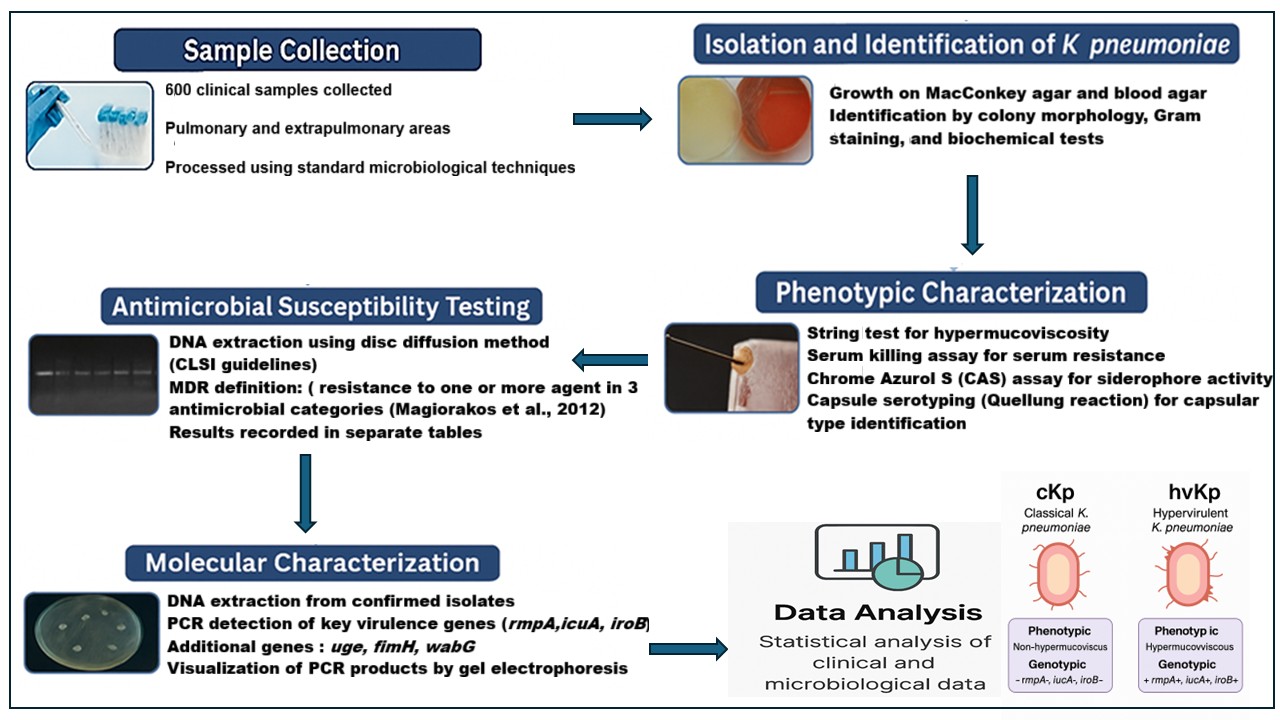

Supplement: SUPPLEMENTARY FIGURE S1 — Flow chart summarizing the methodology for isolation, characterization, and analysis of K. pneumoniae isolates. A total of 600 clinical samples from pulmonary and extra-pulmonary infections were collected and processed using standard microbiological techniques. K. pneumoniae isolates were identified based on colony morphology, Gram staining, and biochemical testing. Phenotypic characterization included the string test for hypermucoviscosity, serum killing assay for serum resistance, Chrome Azurol S (CAS) assay for siderophore activity, and capsule serotyping by the Quellung reaction. Antimicrobial susceptibility was determined by the disk diffusion method according to CLSI guidelines, and multidrug resistance (MDR) was defined per Magiorakos et al. (2012). Molecular characterization involved PCR detection of virulence-associated genes (rmpA, iucA, iroB, uge, fimH1, and wabG). Statistical analysis integrated clinical, phenotypic, and genotypic data to differentiate classical (cKp) and hypervirulent (hvKp) isolates. [file Image_1.JPEG]

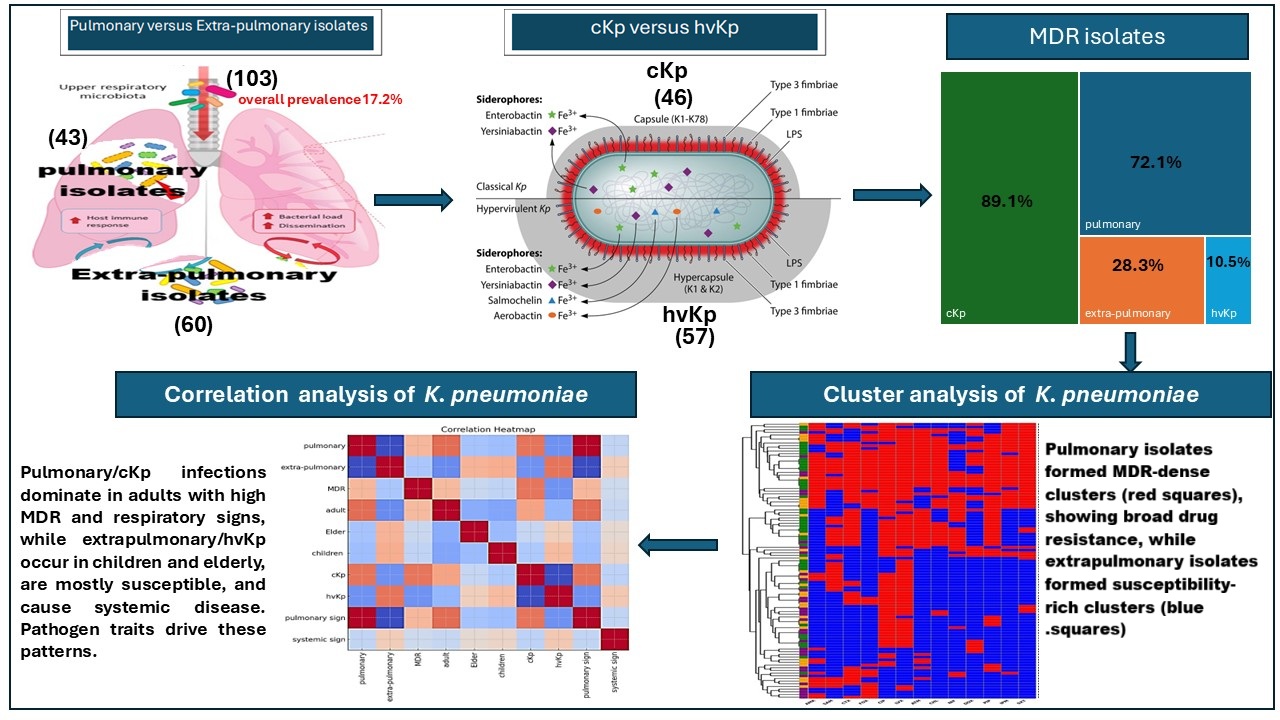

Supplement: SUPPLEMENTARY FIGURE S2 — Comparative analysis of pulmonary and extra-pulmonary K. pneumoniae isolates and their phenotypic, molecular, and resistance profiles. A total of 103 K. pneumoniae isolates were recovered, including 43 pulmonary and 60 extra-pulmonary isolates, with an overall prevalence of 17.2%. Among these, 46 isolates were classified as classical (cKp) and 57 as hypervirulent (hvKp). cKp isolates predominated in pulmonary infections, while hvKp isolates were mainly associated with extra-pulmonary infections. The majority of multidrug-resistant (MDR) strains (89.1%) belonged to cKp, particularly from pulmonary sources (72.1%), whereas hvKp isolates showed lower resistance (10.5%). Correlation analysis revealed that pulmonary/cKp infections were linked to adults and MDR phenotypes, while extra-pulmonary/hvKp infections occurred primarily in children and the elderly with systemic manifestations. Cluster analysis showed that pulmonary isolates formed MDR-dense clusters, while extra-pulmonary isolates grouped into susceptibility-rich clusters, underscoring the distinct clinical and resistance patterns between cKp and hvKp. [file Image_2.JPEG]

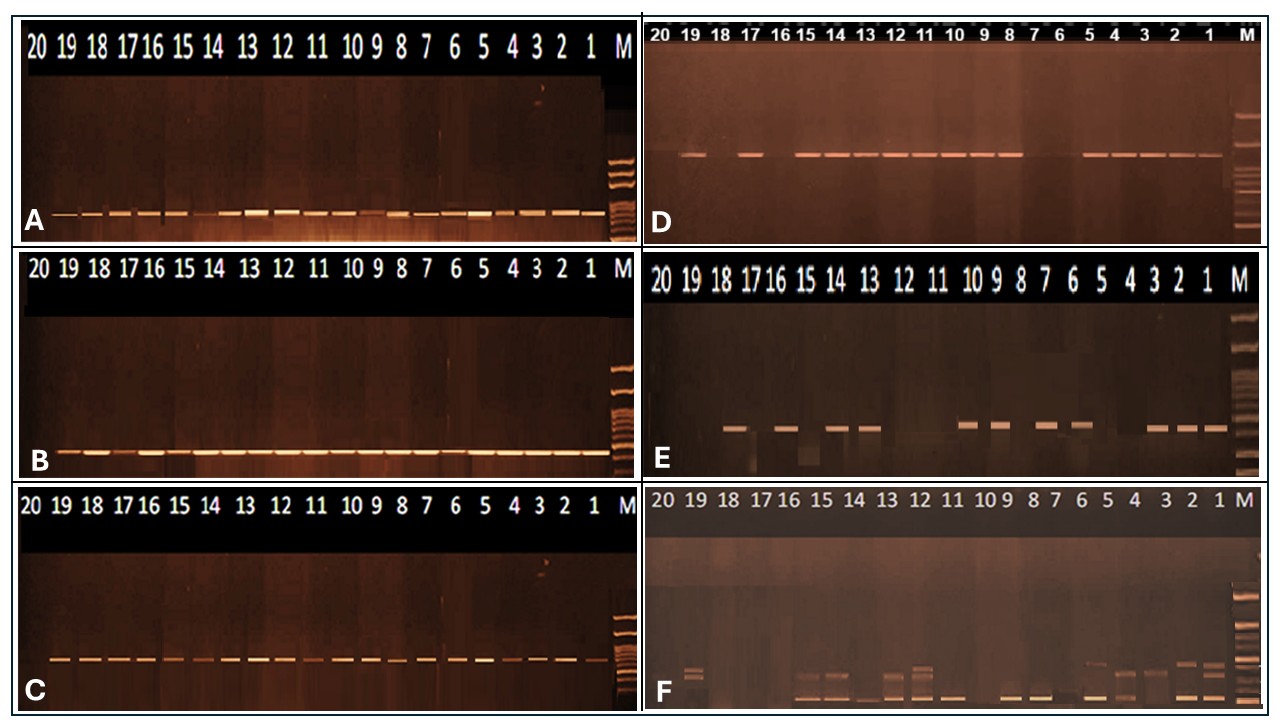

Supplement: SUPPLEMENTARY FIGURE S3 — Gel electrophoresis images showing PCR amplification of virulence and resistance genes among K. pneumoniae isolates. Panels A–C show the detection of virulence-associated genes used to differentiate hypervirulent (hvKp) from classical (cKp) strains, including peg-344 (344 bp) (A), iucA (239 bp) (B), and iroB (585 bp) (C). Panels D–F display the amplification of antimicrobial resistance genes, with blaOXA-48 (763 bp) (D), blaKPC (340 bp) (E), and multiplex PCR detection of, blaCTX-M gene (F). Lane M represents the DNA molecular weight marker, lanes 2–19 correspond to individual K. pneumoniae isolates, lane 1 serves as the positive control, and lane 20 serves as the negative control. The presence of distinct bands confirms the amplification of specific virulence and resistance genes. [file Image_3.JPEG]

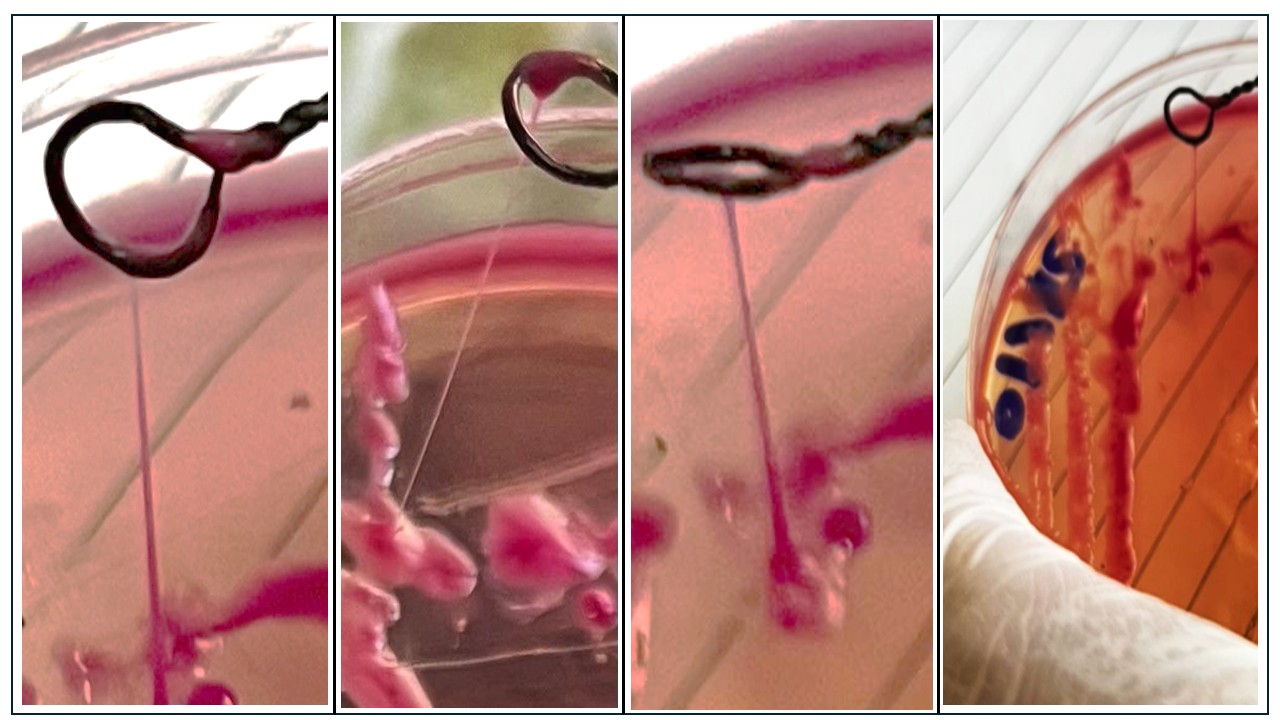

Supplement: SUPPLEMENTARY FIGURE S4 — Hypermucoviscosity assessment using the string test. A positive result is indicated by the formation of a viscous string measuring ≥5 mm when a bacterial colony is stretched upward using an inoculation loop from an agar plate, demonstrating the hypermucoviscous phenotype characteristic of hypervirulent K. pneumoniae (hvKp) isolates. [file Image_4.JPEG]
