# Supplementary material for: Pulmonary and extra-pulmonary infections caused by classical and hypervirulent Klebsiella pneumoniae: a prospective cross-sectional study
Source: Front Microbiol. 2026 Jan 2;16:1707017. doi: 10.3389/fmicb.2025.1707017 (PMC12808397; doi:10.3389/fmicb.2025.1707017)
Supplement: Supplementary file 5 [file Table_1.DOCX]

**Table S: Clinical overview of hospital sites, sample sources, and patient characteristics of all detected K. pneumoniae isolates**

| **Code** | **sample** | **age** | **type** | **symptoms** |
| --- | --- | --- | --- | --- |
| S1 | sputum | adult | cKp1 | tachypnea, tachycardia, productive cough, hypoxia, wheezes |
| S2 | sputum | adult | cKp2 | fever, tachypnea, tachycardia, productive cough, hypoxia, anemia, wheezes |
| S3 | sputum | adult | cKp3 | tachypnea, tachycardia, productive cough, hypoxia, wheezes |
| S4 | sputum | adult | cKp4 | fever, tachypnea, tachycardia, productive cough, hypoxia, cyanosis, pallor |
| S5 | sputum | adult | cKp5 | fever, tachypnea, tachycardia, productive cough, hypoxia, anemia |
| S6 | sputum | children | cKp6 | fever, tachypnea, tachycardia, productive cough, hypoxia, wheezes |
| S7 | sputum | adult | cKp7 | tachypnea, tachycardia, productive cough, hypoxia, hypotension, pallor, wheezes |
| S8 | sputum | adult | cKp8 | fever, tachypnea, tachycardia, productive cough, hypoxia, cyanosis, anemia, pallor |
| S9 | sputum | adult | cKp9 | fever, tachypnea, tachycardia, productive cough, hypoxia, wheezes |
| S10 | sputum | adult | cKp10 | fever, tachypnea, tachycardia, productive cough, hypoxia, anemia |
| S11 | sputum | adult | cKp11 | tachypnea, tachycardia, productive cough, hypoxia, pallor |
| S12 | sputum | adult | cKp12 | fever, tachypnea, tachycardia, productive cough, hypoxia, hypotension, cyanosis, anemia, wheezes |
| S13 | sputum | adult | cKp13 | fever, tachypnea, tachycardia, productive cough, hypoxia, anemia |
| S14 | sputum | adult | cKp14 | fever, tachypnea, tachycardia, productive cough, hypoxia, anemia, pallor |
| S15 | sputum | adult | cKp15 | fever, tachypnea, tachycardia, productive cough, hypoxia, wheezes |
| S16 | sputum | children | cKp16 | fever, tachypnea, tachycardia, productive cough, hypoxia, cyanosis |
| S17 | sputum | adult | cKp17 | tachypnea, tachycardia, productive cough, hypoxia, hypotension |
| S18 | sputum | adult | cKp18 | fever, tachypnea, tachycardia, productive cough, hypoxia, pallor, wheezes |
| S19 | sputum | adult | cKp19 | fever, tachypnea, tachycardia, productive cough, hypoxia |
| S20 | sputum | adult | cKp20 | fever, tachypnea, tachycardia, productive cough, hypoxia, anemia |
| S21 | sputum | adult | cKp21 | fever, tachypnea, tachycardia, productive cough, hypoxia, cyanosis, wheezes |
| S22 | sputum | adult | cKp22 | fever, tachypnea, tachycardia, productive cough, hypoxia |
| S23 | sputum | children | cKp23 | tachypnea, tachycardia, productive cough, hypoxia, hypotension, pallor |
| S24 | sputum | adult | cKp24 | fever, tachypnea, tachycardia, productive cough, hypoxia, anemia, wheezes |
| S25 | sputum | adult | cKp25 | fever, tachypnea, tachycardia, productive cough, hypoxia |
| S26 | sputum | adult | cKp26 | fever, tachypnea, tachycardia, productive cough, hypoxia, cyanosis |
| S27 | sputum | elder | cKp27 | tachypnea, tachycardia, productive cough, hypoxia, pallor |
| S28 | sputum | adult | cKp28 | fever, tachypnea, tachycardia, productive cough, hypoxia, anemia, wheezes |
| S29 | sputum | adult | cKp29 | tachypnea, tachycardia, productive cough, hypoxia, hypotension, pallor |
| S30 | sputum | elder | cKp30 | fever, tachypnea, tachycardia, productive cough, hypoxia, pallor |
| S31 | sputum | adult | cKp31 | fever, tachypnea, tachycardia, productive cough, hypoxia, cyanosis, wheezes |
| S32 | sputum | children | cKp32 | tachypnea, tachycardia, productive cough, hypoxia, hypotension, anemia, pallor |
| S33 | sputum | adult | cKp33 | tachypnea, tachycardia, productive cough, hypoxia |
| S34 | sputum | elder | cKp34 | fever, tachypnea, tachycardia, productive cough, hypoxia |
| S35 | sputum | adult | cKp35 | tachypnea, tachycardia, productive cough, hypoxia, cyanosis, pallor, wheezes |
| S36 | sputum | adult | cKp36 | fever, tachypnea, tachycardia, productive cough, hypoxia, hypotension, anemia |
| S37 | sputum | adult | hvKp1 | fever, tachypnea, tachycardia, productive cough, hypoxia, cyanosis, pallor, wheezes |
| S38 | sputum | elder | hvKp2 | fever, tachypnea, tachycardia, productive cough, hypoxia |
| S39 | sputum | adult | hvKp3 | fever, tachypnea, tachycardia, productive cough, hypoxia, hypotension |
| S40 | sputum | adult | hvKp4 | fever, tachypnea, tachycardia, productive cough, hypoxia, pallor |
| S41 | sputum | adult | hvKp5 | fever, tachypnea, tachycardia, productive cough, hypoxia, anemia, pallor |
| S42 | sputum | adult | hvKp6 | tachypnea, tachycardia, productive cough, hypoxia |
|  | sputum | adult | hvKp7 | tachypnea, tachycardia, productive cough, hypoxia, cyanosis, wheezes |
| B1 | blood | elder | cKp37 | fever, hypotension, pallor |
| B2 | blood | elder | cKp38 | fever |
| B3 | blood | elder | hvKp8 | fever |
| B4 | blood | adult | hvKp9 | fever, cyanosis, pallor |
| B5 | blood | children | hvKp10 | fever, hypotension |
| B6 | blood | elder | hvKp11 | fever, anemia, pallor |
| B7 | blood | elder | hvKp12 | pallor |
| B8 | blood | children | hvKp13 | fever, pallor |
| B9 | blood | elder | hvKp14 | fever, pallor |
| B10 | blood | elder | hvKp15 | anemia, pallor |
| B11 | blood | adult | hvKp16 | fever, hypotension |
| B12 | blood | children | hvKp17 | fever |
| B13 | blood | elder | hvKp18 | fever |
| B14 | blood | children | hvKp19 | pallor |
| B15 | blood | children | hvKp20 | fever, hypotension |
| B16 | blood | children | hvKp21 | fever |
| U1 | urine | children | cKp39 | none |
| U2 | urine | elder | cKp40 | fever |
| U3 | urine | adult | cKp41 | none |
| U4 | urine | elder | cKp42 | hypotension, anemia, pallor |
| U5 | urine | elder | cKp43 | hypotension, anemia |
| U6 | urine | children | cKp44 | fever |
| U7 | urine | children | hvKp22 | fever, cyanosis, anemia |
| U8 | urine | elder | hvKp23 | pallor |
| U9 | urine | children | hvKp24 | fever, hypotension, anemia |
| U10 | urine | children | hvKp25 | fever |
| U11 | urine | children | hvKp26 | fever, pallor |
| U12 | urine | elder | hvKp27 | fever, hypotension, pallor |
| U13 | urine | children | hvKp28 | fever, anemia |
| U14 | urine | elder | hvKp29 | fever, hypotension, pallor |
| U15 | urine | children | hvKp30 | fever, cyanosis |
| U16 | urine | children | hvKp31 | fever, hypotension |
| U17 | urine | elder | hvKp32 | none |
| U18 | urine | elder | hvKp33 | fever |
| U19 | urine | elder | hvKp34 | fever, pallor |
| C1 | csf | children | hvKp35 | fever, hypotension, pallor |
| C2 | csf | children | hvKp36 | none |
| C3 | csf | adult | hvKp37 | fever, cyanosis, anemia |
| C4 | csf | children | hvKp38 | fever, hypotension, pallor |
| C5 | csf | elder | hvKp39 | none |
| C6 | csf | children | hvKp40 | fever |
| C7 | csf | children | hvKp41 | fever, pallor |
| C8 | csf | elder | hvKp42 | fever, hypotension, anemia |
| C9 | csf | adult | hvKp43 | none |
| C10 | csf | elder | hvKp44 | fever, pallor |
| C11 | csf | children | hvKp45 | fever |
| SF1 | synovial fluid | elder | hvKp46 | fever, anemia |
| SF2 | synovial fluid | children | hvKp47 | fever, hypotension |
| SF3 | synovial fluid | children | hvKp48 | fever, cyanosis, anemia |
| SF4 | synovial fluid | elder | hvKp49 | fever, hypotension |
| SF5 | synovial fluid | adult | hvKp50 | fever |
| SF6 | synovial fluid | children | hvKp51 | fever, pallor |
| SF7 | synovial fluid | elder | cKp45 | fever |
| SF8 | synovial fluid | children | cKp46 | fever, pallor |
| AF1 | aspirate fluid | adult | hvKp52 | fever, hypotension |
| AF2 | aspirate fluid | elder | hvKp53 | fever, anemia |
| AF3 | aspirate fluid | children | hvKp54 | fever, pallor |
| AF4 | aspirate fluid | elder | hvKp55 | fever |
| AF5 | aspirate fluid | children | hvKp56 | fever, hypotension |
| AF6 | aspirate fluid | children | hvKp57 | fever, anemia |
